# Astragaloside IV alleviated bone loss in mice with ovariectomy-induced osteoporosis via modulating gut microbiota and fecal metabolism

**DOI:** 10.3389/fphar.2025.1548491

**Published:** 2025-04-03

**Authors:** Huichao Wang, Zhongyue Huang, Guangnan Chen, Yang Li, Youwen Liu, Huijie Gu, Yujing Cao

**Affiliations:** ^1^ School of Osteopathy, Henan University of Chinese Medicine, Zhengzhou, Henan, China; ^2^ Luoyang Orthopedic-Traumatological Hospital of Henan Province (Henan Provincial Orthopedic Hospital), Orthopedic Institute of Henan Province, Luoyang, Henan, China; ^3^ Department of Orthopedics, Minhang Hospital, Fudan University, Shanghai, China; ^4^ Emergency Trauma Center, Henan Provincial Hospital of Traditional Chinese Medicine, Zhengzhou, Henan, China

**Keywords:** astragaloside IV, osteoporosis, gut microbiota, metabolism, intestinal barrier, oxidative stress

## Abstract

**Background:**

Astragaloside IV (AS-IV) is one of the most potent components of Astragalus. It has been reported to promote bone formation and inhibit osteoclastogenesis, suggesting its potential as a candidate for the prevention and treatment of postmenopausal osteoporosis (PMOP). The gut microbiota may play a crucial role in mediating the effects of AS-IV.

**Objective:**

To investigate the impact of gut microbiota on the efficacy of AS-IV in treating PMOP.

**Methods:**

Mice were randomly divided into three groups: Sham, ovariectomy (OVX), and AS-IV-treated OVX group (80 mg/kg). Bone loss was evaluated using Micro-CT and histopathology. Immunohistochemistry assessed specific bone markers. Inflammatory levels were measured by enzyme-linked immunosorbent assay (ELISA). Intestinal barrier function was examined via colonic histopathology and immunohistochemistry. Gut microbiota composition was analyzed by 16S rDNA sequencing, while metabolomic profiling identified key metabolites. Correlation analysis was performed to explore relationships between differential bacteria, key metabolites, and bone loss.

**Results:**

AS-IV improved the femur microarchitecture and modulated bone turnover in OVX mice. AS-IV treatment strengthened the intestinal barrier function and decreased gut permeability. This compound reduced colonic oxidative stress and serum and bone marrow inflammatory cytokine production. 16S rDNA sequencing revealed that AS-IV modulated the gut microbiota composition, while metabolomic analysis showed its effects on pathways related to hormone biosynthesis, D-amino acid metabolism, and galactose metabolism.

**Conclusion:**

This study provides new insights into the use of AS-IV for treating PMOP, highlighting the gut microbiota and its metabolites as key regulatory factors in AS-IV’s therapeutic effects.

## Graphical Abstract

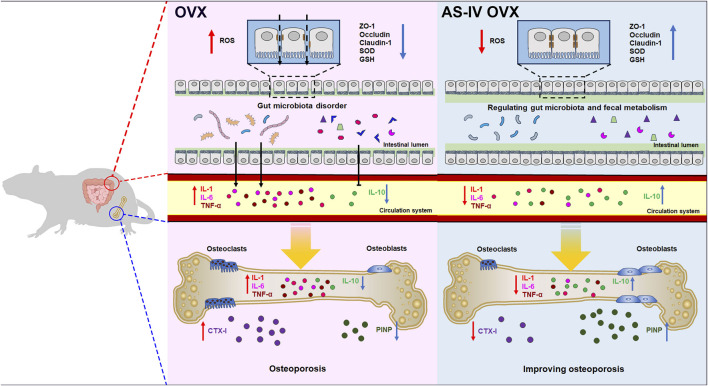

## Introduction

Postmenopausal osteoporosis (PMOP) is a prevalent form of primary osteoporosis (POP) caused by decreased estrogen levels (Rosen, 2000). Bisphosphonates, such as alendronate, are currently the most widely used treatments for PMOP. However, their use is limited by potential side effects such as nephrotoxicity, poor patient adherence, various adverse reactions, risk of jaw osteonecrosis, and high costs (Reid and Billington, 2022; LeBoff et al., 2022). Therefore, Due to these limitations, there is a strong focus on further understanding the pathophysiology of PMOP and developing safer, more effective therapeutic options to meet clinical needs.

In the pathogenesis of PMOP, estrogen deficiency-induced pro-inflammatory phenotype and oxidative stress are key contributors to bone loss (Li et al., 2020). Specifically, the decline in estrogen levels leads to elevated levels of inflammatory mediators, including interleukin-1 (IL-1), interleukin-6 (IL-6), and tumor necrosis factor-alpha (TNF-α), which not only promote osteoclastogenesis but also enhance bone resorption (Zhu et al., 2018). Furthermore, the absence of estrogen triggers oxidative stress, evidenced by reduced antioxidant enzyme activity in ovariectomized (OVX) animal models and postmenopausal women (Zhang et al., 2023a). The excessive accumulation of reactive oxygen species (ROS) further amplifies osteoclast activity by stimulating cytokine production, including IL-1, IL-6 and IL-7 (Bonaccorsi et al., 2018). Additionally, oxidative stress and elevated ROS levels reduce osteoblast lifespan in OVX and aged mice by inducing apoptosis (Almeida et al., 2007). Thus, targeting chronic inflammation and oxidative stress is crucial in the prevention and treatment of PMOP.

Natural products play a crucial role in drug discovery (Gray et al., 2012), yet the unclear mechanisms of many natural compounds limit their therapeutic development (Soares, 2010). Astragaloside IV (AS-IV), a triterpenoid saponin extracted and purified from Astragalus membranaceus, exhibits a wide range of pharmacological effects, including antioxidant, cardioprotective, anti-inflammatory, antiviral, antibacterial, antifibrotic, antidiabetic, and immunomodulatory activities (Chen et al., 2020; Shi et al., 2021; Zhong et al., 2022). *In vitro* studies have demonstrated that AS-IV reduces levels of pro-inflammatory mediators, including myeloperoxidase (MPO), TNF-α, IL-1β, IL-6, and nitric oxide (NO) (Wu and Chen, 2019). AS-IV also targets PRDX6 to inhibit PLA2 activity, reducing ROS production and alleviating oxidative stress damage (Cheng et al., 2023). Additionally, in a lipopolysaccharide (LPS)-induced acute respiratory distress syndrome model in pulmonary endothelial cells, AS-IV was shown to reduce inflammation and autophagy by inhibiting oxidative stress and inflammatory responses (Zhang et al., 2020). Recent studies have further revealed that AS-IV promotes osteoblastogenesis and inhibits osteoclastogenesis (Li et al., 2015; Bian et al., 2011), indicating its potential as an anti-osteoporotic agent for preventing and treating PMOP. These findings highlight the therapeutic potential of AS-IV in addressing both inflammatory and oxidative stress pathways in osteoporosis.

Despite these broad therapeutic effects, AS-IV suffers from poor oral bioavailability and membrane permeability, limiting its ability to enter the bloodstream and brain (Li et al., 2023). Researchers hypothesize that key regulatory factors mediate the therapeutic efficacy of AS-IV in treating postmenopausal osteoporosis (PMOP). According to the “gut-bone axis” hypothesis, there is a strong connection between gut microbiota and PMOP (Hansen and Frost, 2022), and beneficial changes in the gut microbiome can improve bone metabolism by reducing systemic inflammation and enhancing antioxidant activity (Li et al., 2019; Tanabe et al., 2019). It is speculated that AS-IV may exert its bone-protective effects by modulating gut microbiota and microbial products, despite its low absorption rate. This study systematically investigates the efficacy of AS-IV in treating PMOP and evaluates its impact on gut microbiota composition and intestinal metabolic pathways in an OVX mouse model.

## Methods and materials

### AS-IV preparation

AS-IV with a purity of ≥98% and a molecular weight of 784.97 was purchased from Yuan Ye Biotech, Shanghai, China.

### OVX mouse model

A total of 30 six-week-old female C57BL mice were obtained from the Model Animal Research Center of Shanghai, China, for use in an OVX mouse model. Surgical procedures were performed under sodium pentobarbital anesthesia, with mice undergoing either a sham operation or bilateral ovariectomy following previously described methods (Boyd et al., 2006). The mice were randomly assigned to one of three groups (n = 10 per group): Group I (Sham group), where the mice underwent a sham operation; Group II (OVX group), consisting of mice that underwent ovariectomy; and Group III (AS_OVX group), consisting of OVX mice treated with AS-IV. All mice were housed under standard laboratory conditions with *ad libitum* access to food and water. The study duration was 12 weeks, with the AS_OVX group receiving daily oral AS-IV starting in the second week, while the Sham and OVX groups were administered distilled water.

### Sample collection

Sample collection was performed 12 weeks post-surgery. Fecal samples were collected individually from each mouse and stored at −80°C for subsequent gut microbiota and metabolomic analyses. At the end of the experiment, blood samples were collected following a 12-h fasting period. The mice were anesthetized using sodium pentobarbital, and blood was collected from the retro-orbital venous plexus. The serum was isolated and stored at −80°C for future biochemical assessments. Bone samples were also collected: the right femur was dissected, and the bone marrow cavity was flushed with pre-cooled PBS until the bone appeared white. The left femur was dissected and placed in 10% neutral buffered formalin for subsequent micro-CT and histopathological evaluations. Additionally, organs such as the uterus, spleen, and thymus were weighed and stored at −80°C for future investigations. For colonic tissue, a segment of 1–1.5 cm was excised, rinsed with PBS, and divided, with one portion frozen at −80°C and the remaining tissue fixed in 10% neutral buffered formalin for further examination.

### Micro-CT analysis

Femur samples were fixed in 4% paraformaldehyde (PFA) for 24 h prior to analysis. Micro-computed tomography (micro-CT) scans were performed using a Bruker micro-CT system (Bruker Corp., Billerica, MA, USA). Quantitative analysis of the reconstructed bone images was conducted to assess key parameters, including bone mineral density (BMD), bone volume fraction (BV/TV), bone surface area to total volume ratio (BS/TV), trabecular number (Tb·N), trabecular thickness (Tb·Th), and trabecular separation (Tb·Sp). These metrics were used to evaluate bone microarchitecture.

### ROS detection

Reactive oxygen species (ROS) detection was performed using MitoSOX Red and CellROX Green fluorescent dyes to detect mitochondrial and cytoplasmic ROS, respectively (Invitrogen, CA, United States). Colon sections were exposed to 5 µM of each dye and incubated for 30 min at 37°C. Images were captured using an LSM T-PMT confocal microscope (Zeiss).

### Assessment of oxidative stress markers

Oxidative stress markers were assessed by measuring reduced glutathione (GSH) using a commercial kit (Servicebio, G4305, Wuhan, Hubei, China). GSH activity was expressed in μM per kilogram of tissue. Superoxide dismutase (SOD) activity was determined using the xanthine oxidase assay kit (Servicebio, G4306, Wuhan, Hubei, China).

### HE staining

Tissue fixation was performed by placing femur and colon samples from all groups in 4% paraformaldehyde (Servicebio, Wuhan, Hubei, China) for 24 h. Femurs were decalcified using 12.5% EDTA (Servicebio) for 3-4 weeks. Decalcified samples were then embedded in paraffin, and 5 µm thick tissue sections were prepared. Hematoxylin and eosin (H&E) staining was applied to both femur and colon sections for histological analysis.

### AB-PAS staining

Colon tissue sections were stained using the Alcian Blue-Periodic Acid Schiff (AB-PAS) method to assess pathological changes. After dehydration through an ethanol gradient, sections were stained with AB-PAS solution. Under microscopy, acidic mucus appears blue, neutral mucus red, and mixed mucus purple-red. Staining followed previously established protocols (Guo et al., 2023).

### TRAP staining

Femur sections were treated using tartrate-resistant acid phosphatase (TRAP) stain to identify osteoclasts. TRAP specifically labels osteoclasts due to their high acid phosphatase activity. Quantification of osteoclasts was performed using ImageJ software (National Institutes of Health, Bethesda, MD, United States), a widely used tool for image processing and analysis.

### Immunohistological analysis

Tissue sections were equilibrated in 0.1 M Tris-buffered saline for 10 min. Blocking was performed by incubating the sections in phosphate-buffered saline (PBS) containing 10% normal goat serum for 1 h. For primary antibody step, femur sections were incubated with anti-osteocalcin (OCN) antibody (1:200 dilution, Proteintech, 23418-1-AP, United States), while colon sections were incubated with anti-Occludin, anti-ZO-1, and anti-Claudin 1 antibodies (AF0321 diluted 1:200 and AF6504 diluted 1:50, Beyotime, China). All sections were incubated with the primary antibodies overnight at 4°C. Horseradish peroxidase (HRP)-conjugated secondary antibodies (1:50 dilution, Beyotime, A0208, China), were applied for 1 h at room temperature. Visualization of Occludin, ZO-1, and Claudin 1 was achieved using 3,3′-diaminobenzidine (DAB). Quantitative analysis of positive signals was performed using ImageJ software (NIH, United States) in six randomly selected high-power fields (HPF) per sample to assess the expression of these markers.

### Biochemical analysis

Biochemical analyses were conducted to measure the expression levels of procollagen type I N-terminal propeptide (PINP) and C-terminal telopeptide of type I collagen (CTX-I) in mice. Inflammatory cytokines, including interleukin-1β (IL-1β), interleukin-6 (IL-6), tumor necrosis factor-α (TNF-α), and interleukin-10 (IL-10), were also measured in bone marrow tissue and serum. All measurements were carried out using enzyme-linked immunosorbent assay (ELISA) kits, according to the manufacturer’s protocols. ELISA kits were obtained from DO Biological (Shanghai, China).

### 16S rRNA sequencing and bioinformatics analysis

Total genomic DNA was extracted using a DNA extraction kit following the manufacturer’s instructions. PCR amplification was performed using barcoded primers and Tks Gflex DNA polymerase (Takara) with the extracted genomic DNA as the template. The V3-V4 variable region of the 16S rRNA gene was selected for bacterial diversity analysis, amplified using universal primers F343/R798 with a dual-index method. Sequencing was conducted on the Illumina NovaSeq6000 platform, generating paired-end 250-base reads for each sample. Sequencing services were provided by Illumina (San Diego, CA, United States) and OE Biotech (Shanghai, China).

### Fecal metabolomics analysis and metabolites identification

Fecal metabolomics analysis was conducted using gas chromatography-mass spectrometry (GC-MS) with an Agilent 7890B GC system and Agilent 5977B MSD system (Shanghai Lu-Ming Biotech Corp., Shanghai, China). Data analysis included partial least-squares discriminant analysis (PLS-DA) and orthogonal PLS-DA (OPLS-DA). Differential metabolites were identified based on log2(FC) >2 and p-value <0.05. Metabolic pathway enrichment analysis was performed using MetaboAnalyst software with the KEGG database.

### Statistical analysis

The data were processed using GraphPad Prism 8 software and expressed as mean ± standard deviation (SD). Statistical comparisons were made using one-way ANOVA or Student’s t-test, depending on the context. Correlation analyses utilized Pearson’s correlation coefficient, and chi-square tests were used for comparing categorical data. A p-value of less than 0.05 was considered statistically significant, ensuring rigorous evaluation of the experimental results and their implications.

## Results

### AS-IV improved the femur microarchitecture in OVX mice

**FIGURE 1:**
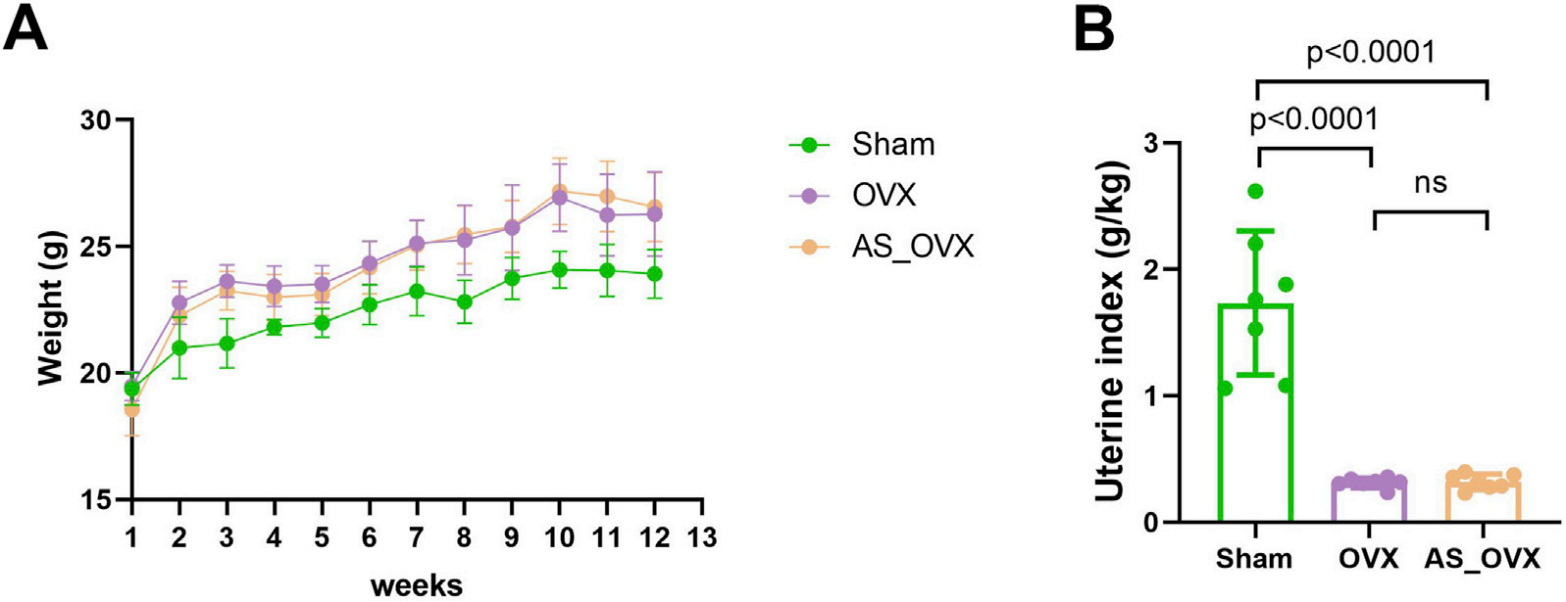
AS-IV treatment regulated phenotypic features in OVX mice. **(A)** Changes in body weight of experimental animals over the course of the experimental period. **(B)** Differences in uterine index (uterine weight/body weight) among groups.

As exhibited in Figure 1A, the OVX group and AS-IV group of mice showed a greater increase in body weight compared to the sham-operated (Sham) group. Additionally, the uterine index (uterine weight/body weight) markedly decreased in the OVX group, confirming the successful establishment of the ovariectomy model. AS-IV treatment did not increase the uterine index compared to OVX group (Figure 1B). Overall, AS-IV had no significant effect on the estrogen deficiency-induced weight gain or uterine atrophy in the treated mice.

**FIGURE 2:**
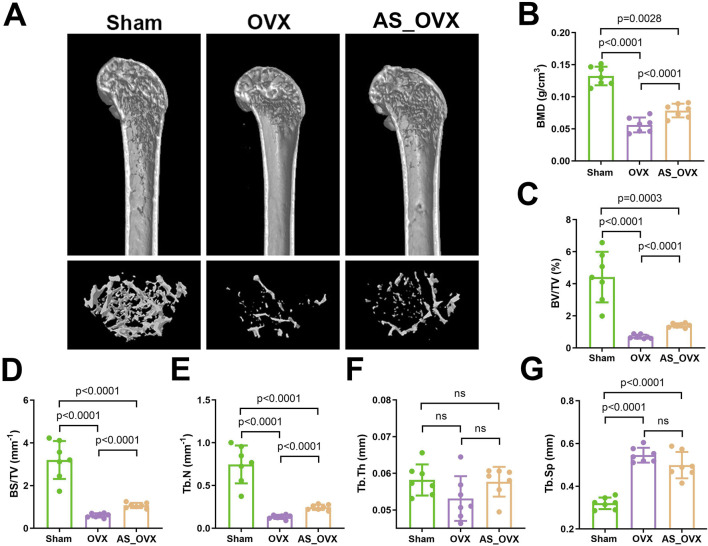
AS-IV treatment improved the femur microarchitecture in OVX mice. **(A)** the femur distal metaphyseal region of rats was analyzed using micro-computed tomography (CT). **(B–G)** Comparison of BMD, BV/TV, BS/TV, Tb.N, Tb.Th, and Tb.Sp between groups. Data are expressed as mean ± standard deviation. (n = 7). BMD, bone mineral density; BV/TV, bone volume/total volume; BS/TV, bone surface area/total volume; Tb.N, trabecular number; Tb.Th, trabecular thickness; Tb.Sp, trabecular separation.

Micro-CT scanning was used to reconstruct the trabecular distribution in the femurs of different groups, with the results were shown in Figure 2A. In the Sham group, the trabeculae were uniformly distributed, well-organized, and formed a compact network structure. In contrast, the OVX group exhibited significant disruption of bone microarchitecture, with noticeable voids at the trabecular connections. The AS_OVX group showed a reduction in marrow cavity size, partial restoration of trabecular number, width, length, and shape, and an increase in the density of the trabecular network.

Quantitative morphometric measurements based on scanning images (Figure 2B–G) revealed significant changes in bone parameters in the OVX group. BMD, BV/TV, BS/TV, and Tb.N were significantly reduced, while Tb.Sp increased. In the AS-IV-treated group, BMD, BV/TV, BS/TV, and Tb.N were significantly higher, and Tb.Sp was lower compared to the OVX group. However, these parameters in the AS-IV group were still lower than in the Sham group, with Tb.Sp remaining higher. These findings suggest that AS-IV treatment partially improved the femoral microstructure in OVX mice.

### AS-IV treatment modulated bone turnover induced by estrogen deficiency

**FIGURE 3:**
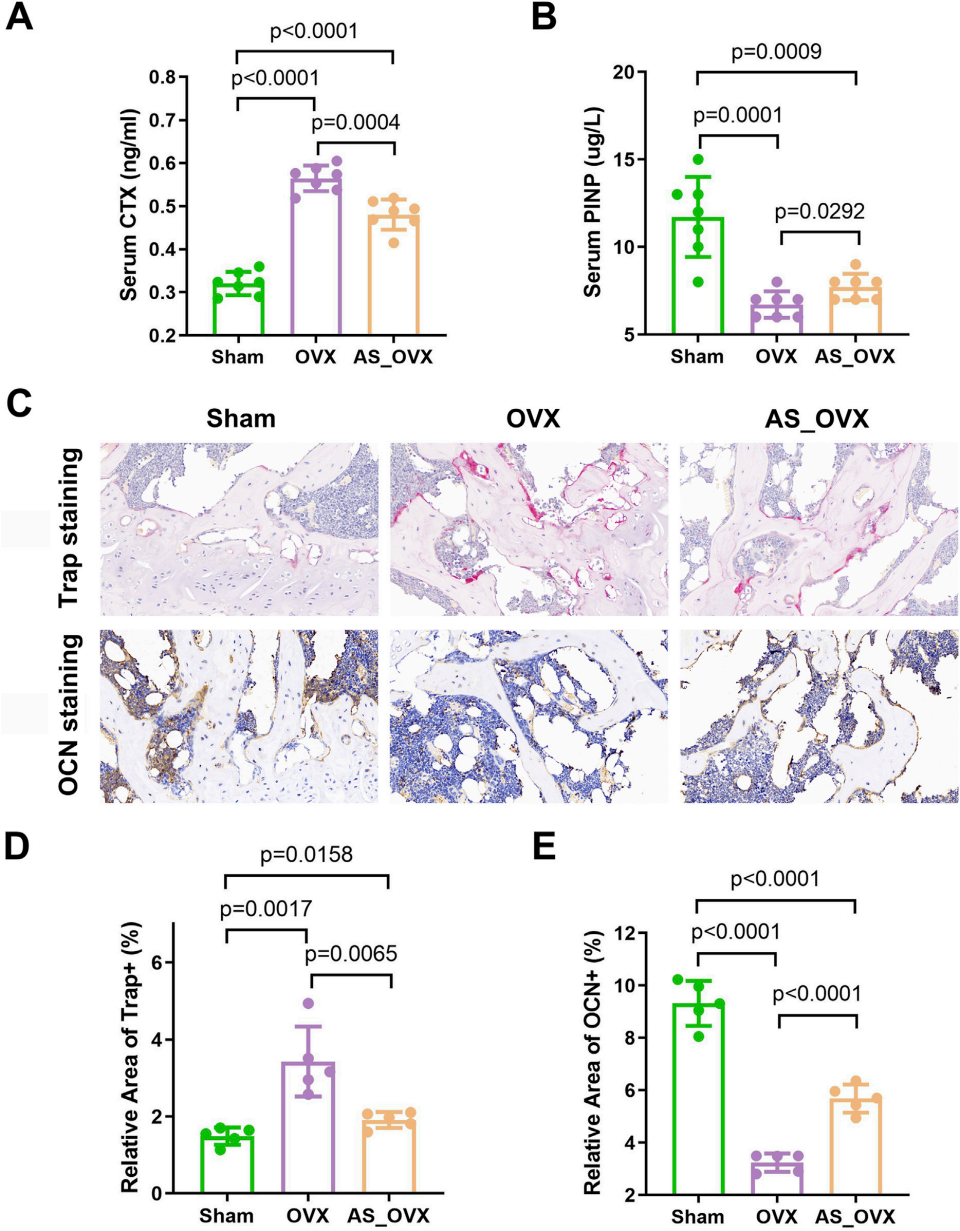
AS-IV treatment improved expression of bone turnover markers in ovariectomized mice. **(A, B)** The changes of serum CTX and PINP were observed among the groups. (n = 7). **(C)** Representative images of tartrate-resistant acid phosphatase (TRAP) staining and osteocalcin (OCN) staining in femurs (scale bars, 50 μm). **(D)** Quantification of relative area of Trap^+^ cells in femurs (n = 5). **(E)** Quantification of relative area of OCN^+^ cells in femurs (n = 5). Data are expressed as mean ± standard deviation. CTX, C-telopeptide of type I collagen; PINP, N-terminal propeptide of type I procollagen.

Serum markers of bone metabolism were assessed by measuring the bone formation marker procollagen type I N-terminal propeptide (PINP) and the bone resorption marker C-terminal telopeptide of type I collagen (CTX-1). In the OVX group, serum PINP levels were significantly reduced, while CTX-1 levels were significantly elevated compared to the Sham group. AS-IV treatment significantly increased PINP levels and decreased CTX-1 levels compared to the OVX group. However, despite these improvements, PINP remained lower and CTX-1 higher in the AS-IV group compared to the Sham group (Figure 3A, B).

TRAP staining revealed a significant increase in TRAP-positive areas, indicating elevated osteoclast activity in femur sections from OVX mice (Figure 3C, D). AS-IV treatment reduced the TRAP-positive areas compared to the OVX group, suggesting a decrease in osteoclast activity. OCN staining showed a significant reduction in OCN-positive areas, reflecting decreased osteoblast activity in OVX mice. AS-IV treatment increased the OCN-positive areas, indicating enhanced osteoblast activity (Figure 3C, E). However, despite these positive effects on bone metabolism, the AS-IV-treated group still had lower PINP levels and higher CTX-1 levels compared to the Sham group, indicating incomplete restoration of bone metabolism to normal levels.

In conclusion, AS-IV treatment can prevent OVX-induced bone loss by decreasing bone resorption and increasing bone formation.

### AS-IV treatment strengthened the intestinal barrier function and decreased gut permeability

**FIGURE 4:**
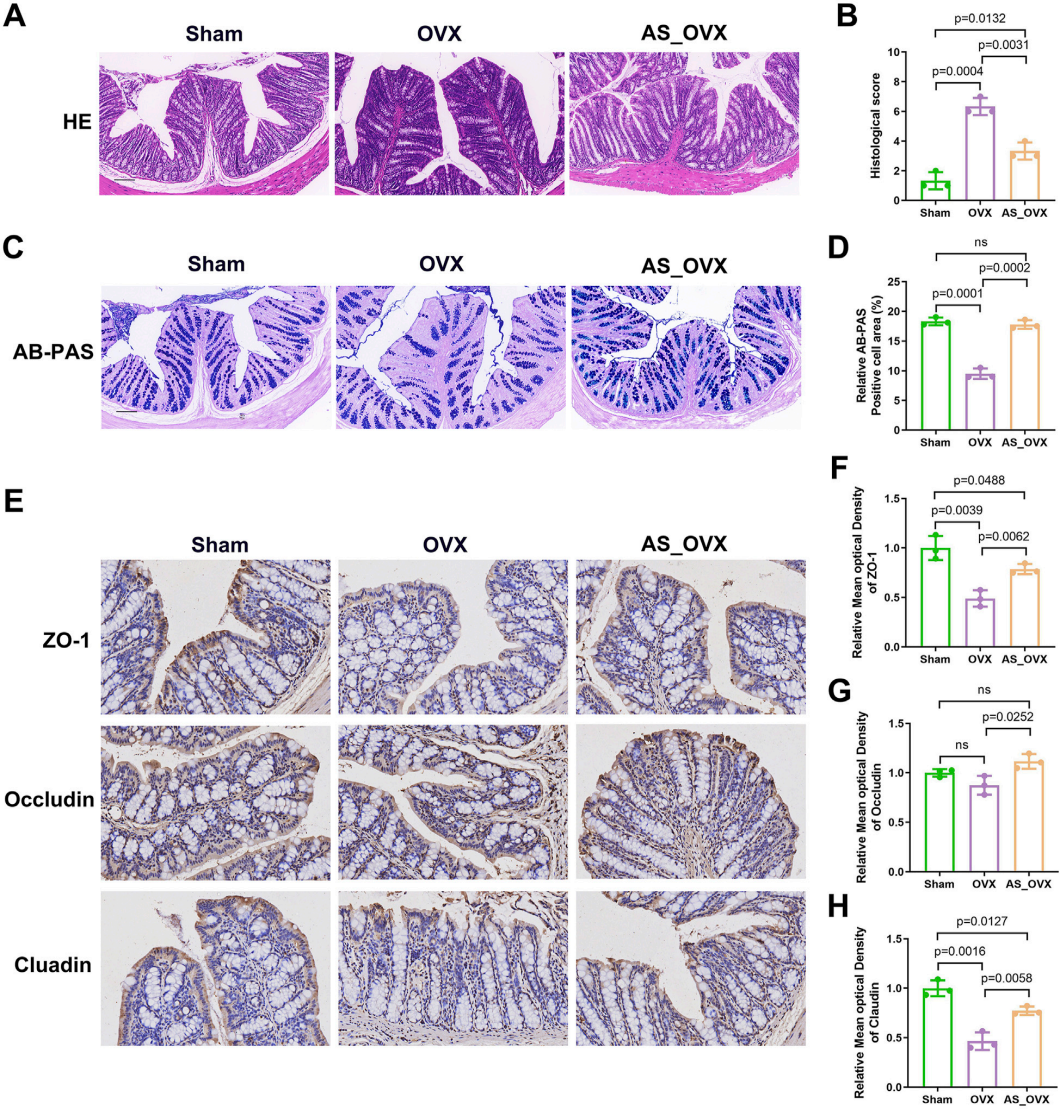
The effects of astragaloside IV (AS-IV) on intestinal barrier integrity in OVX mice. **(A)** Representative images of Hematoxylin and eosin (H&E) staining in colon sections. Scale bar: 50 µm. **(B)** Intestinal morphology studies measure cumulative scores including inflammation infiltration and crypt damage and goblet cells damage. The scoring criteria are shown in Supplementary Table S1. **(C)** Representative images of AB-PAS staining. Scale bar: 50 µm. **(D)** The quantification of goblet cells in AB -PAS staining (n = 3). **(E)** Immunohistochemistry analysis of ZO-1, Occludin and Claudin (scale bar, 50 µm) (n = 3). **(F–H)** Immunohistochemical analysis (n = 3).

H&E staining revealed impaired intestinal structural integrity in OVX mice, characterized by mucosal damage, distorted crypt morphology, and inflammatory cell infiltration. AS-IV treatment significantly alleviated these pathological changes compared to the OVX group (Figures 4A, B). AB-PAS staining showed mucus-secreting goblet cells stained purple (Figure 4C). The OVX group exhibited marked goblet cell atrophy, with fewer goblet cells in the crypts and reduced mucus production. AS-IV treatment improved goblet cell atrophy and increased the goblet cell area in the crypts (Figure 4D). The AB-PAS-positive cell area was markedly decreased in the OVX group (p < 0.0001), while AS-IV treatment increased the AB-PAS-positive cell area (p < 0.05, Figure 4D).

Immunohistochemical analysis showed a significant reduction in the expression of tight junction proteins ZO-1 and claudin-1 in the colon of OVX group compared to the Sham group. AS-IV treatment significantly enhanced the levels of ZO-1 and claudin-1 compared to the OVX group, although the levels remained lower than in the Sham group. Quantitative analysis of the immunohistochemical images indicated that the decreased expression of ZO-1 and claudin-1 in the OVX group might contribute to increased intestinal permeability (Figures 4E–H). AS-IV treatment not only increase the occludin expression along with ZO-1 and claudin compared to OVX group (Figures 4E–H). These results indicated that AS-IV treatment may help maintain epithelial integrity by preserving TJ protein expression in the colon.

### AS-IV treatment alleviated oxidative stress and inflammatory cytokine production induced by estrogen deficiency

**FIGURE 5:**
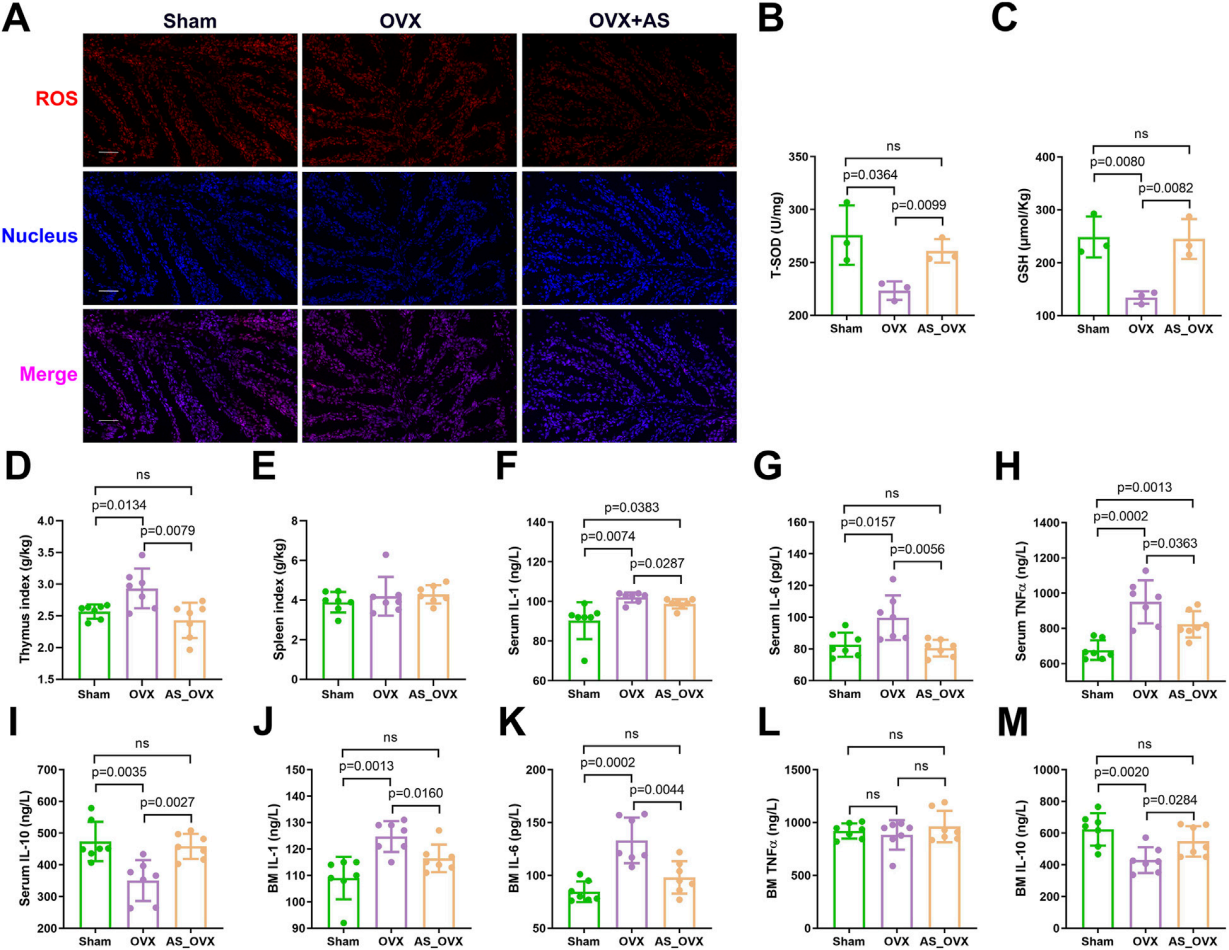
AS-IV treatment alleviated oxidative stress and inflammatory cytokine production induced by estrogen deficiency. **(A)** Images of intestinal reactive oxygen species (ROS) staining. Scale bar: 50 µm. **(B, C)** Total superoxide dismutase (T-SOD) activity and reduced glutathione (GSH) concentrations in intestinal tissues (n = 3). **(D, E)** Thymus and spleen index (organ weight/body weight) (n = 7). **(F–I)** The inflammatory IL-1β, IL-6, TNF-α, and IL-10 levels in serum (n = 7). **(J–M)** The inflammatory IL-1β, IL-6, TNF-α, and IL-10 levels in bone marrow (BM) (n = 7).

The aim of this study was to evaluate the effects of AS-IV on intestinal oxidative stress in OVX group. Oxidative stress markers were assessed by measuring reactive oxygen species (ROS), reduced glutathione (GSH), and superoxide dismutase (SOD) activity. A significant accumulation of ROS was detected in the intestines of OVX mice. AS-IV treatment significantly reduced ROS accumulation (Figure 5A). Total SOD (T-SOD) and GSH levels were notably decreased in the OVX group when compared to the Sham group. However, AS-IV treatment significantly increased T-SOD and GSH levels compared to the OVX group (Figures 5B, C).

In this study, thymus index (thymus weight/body weight) was significantly elevated in OVX group, indicating the potential presence of systemic inflammation. AS-IV treatment significantly reduced the thymus index. However, no significant differences were observed in the spleen index (spleen weight/body weight) among the three groups (Figure 5D, E). Serum inflammatory cytokine analysis revealed that pro-inflammatory cytokines such as IL-1, IL-6, and TNF-α were significantly upregulated in the OVX group compared to the sham-operated (Sham) group, while the anti-inflammatory cytokine IL-10 was downregulated (Figure 5F–I). AS-IV treatment significantly suppressed the levels of pro-inflammatory cytokines and increased IL-10 levels.

Bone marrow cytokine analysis using ELISA yielded results consistent with the serum findings, showing significant differences in IL-1, IL-6, and IL-10 levels between the OVX and AS-IV treatment groups (Figure 5J–M). Specifically, AS-IV resulted in a significant reduction in IL-1 and IL-6 levels and an increase in IL-10 levels (p < 0.05), though TNF-α levels did not differ significantly between the OVX and AS-IV groups.

In serum, IL-1 and TNF-α levels were significantly elevated in the OVX group compared to the Sham group, but other inflammatory cytokines did not exhibit significant differences (p > 0.05). In conclusion, AS-IV treatment significantly reduced inflammatory cytokines in both serum and bone marrow and attenuated oxidative stress within the colon (p < 0.05).

### AS-IV treatment regulated gut microbiota

**FIGURE 6:**
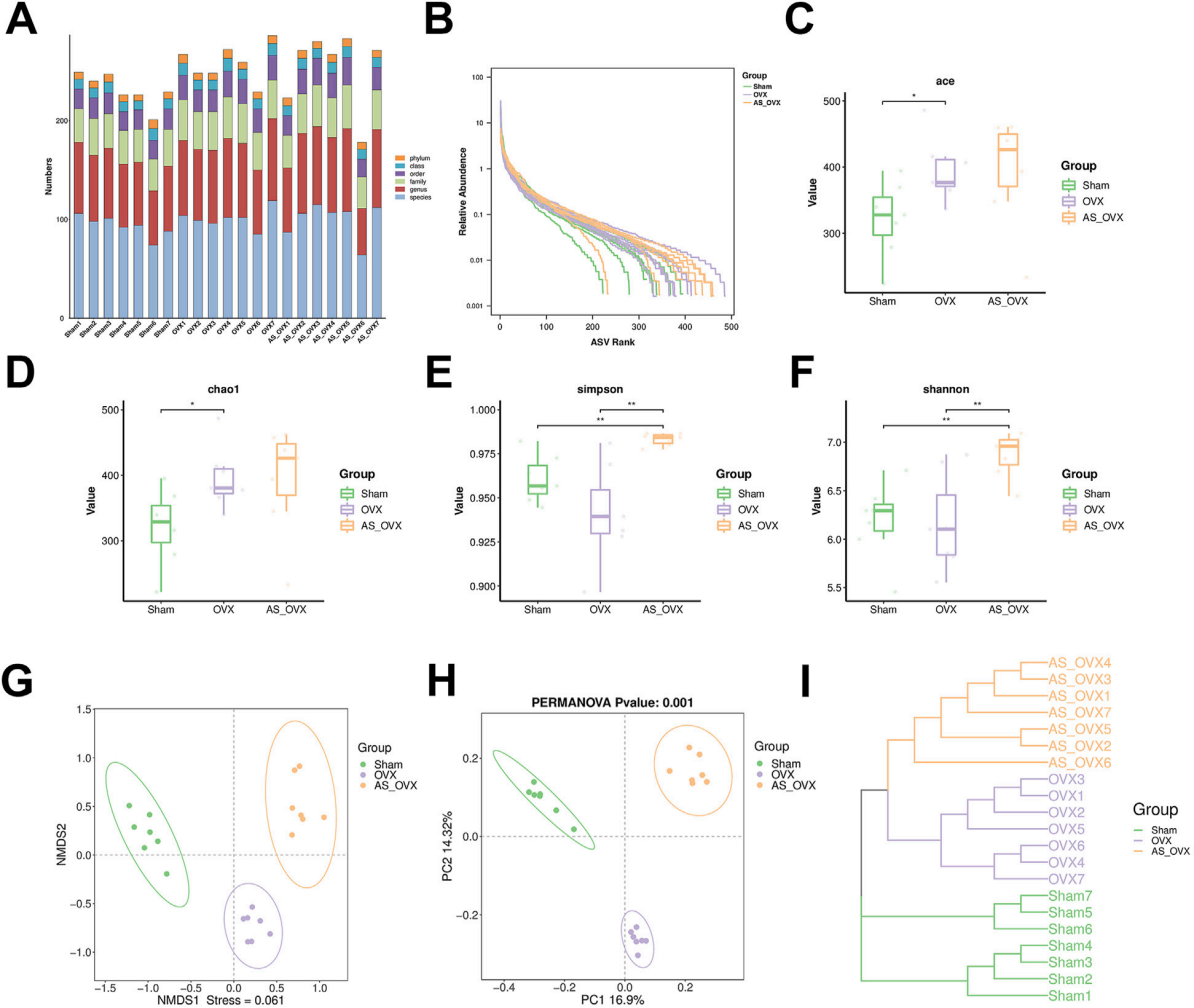
AS-IV treatment improved the gut microbiota disorder in ovariectomized mice. **(A)** The microbial community structure of each sample. Rank abundance curve **(B)**, ACE estimator **(C)**, Cho 1 estimator **(D)**, Simpson index **(E)** and Shannon index **(F)**. **(G, H)** Beta diversity analysis of intestinal microbiota among the four groups using the NMDS and PCoA method. **(I)** Clustering analysis of gut microbiota among samples. Data are shown as the mean ± SD. *p < 0.05, **p < 0.01.

Changes in the gut microbiota were analyzed to assess the impact of (AS-IV treatment on the gut microbiota of OVX-induced osteoporotic mice. The composition of the gut microbiota in the Sham, OVX, and AS_OVX groups was evaluated. The microbiota community structure in each sample is depicted in Figure 6A, and the ranked abundance curve flattened, indicating sufficient sequencing depth to cover the diversity of transcripts (Figure 6B). Alpha diversity indices showed a slight increase in ACE and Chao1 indices in the OVX group compared to the Sham group (Figures 6C, D). Following AS-IV treatment, these alpha diversity indices further increased, with the Simpson and Shannon indices being significantly higher in the AS_OVX group compared to both the Sham and OVX groups (p < 0.05) (Figures 6E, F). Beta diversity was analyzed using Non-metric Multidimensional Scaling (NMDS) and Principal Coordinates Analysis (PCoA) based on Operational Taxonomic Units (OTUs), to visualize the similarities between microbiota communities. The results demonstrated clear separation between the Sham, OVX, and AS_OVX groups by week 12 post-AS-IV treatment (Figure 6G, H). Hierarchical clustering based on weighted UniFrac distances further confirmed the differences between samples (Figure 6I). The gut microbiota of AS-IV-treated mice displayed significant alterations compared to both the OVX and Sham groups.In conclusion, OVX and AS-IV treatment induced significant changes in the bacterial community, suggesting that AS-IV may improve osteoporosis by modulating the gut microbiota. These findings highlight the potential role of gut microbiota in the therapeutic effects of AS-IV on bone health.

At the phylum level, Firmicutes, Bacteroidota and Desulfobacterota were the dominant bacterial phyla across all groups (Supplementary Figure S1A). At the class level, Bacteroidia, Clostridia, and Bacilli accounted for over 95% of the total microbiota in all groups (Supplementary Figure S1B). At the order level, Bacteroidia, Clostridia and Bacilli were the most abundant (Supplementary Figure S1C). No notable differences were detected in the abundance of Bacteroidota and Firmicutes, nor in the Firmicutes and Bacteroidota ratio (F/B) between the groups (Supplementary Figures S1D–F).

At the family level, the most abundant bacterial families across all groups were Muribaculaceae, Prevotellaceae, and Lachnospiraceae (Supplementary Figure S2A). Compared to the Sham group, the OVX group indicated a significant enrichment in Prevotellaceae, F082 and Tannerellaceae, but a reduction in Muribaculaceae (Supplementary Figures S2B, C, F, H). AS-IV treatment reversed some of bacteria abundances, such as Prevotellaceae and Tannerellaceae (Supplementary Figures S2C, H). In addition, AS-IV group showed significant enrichment in Lactobacillaceae and Rikenellaceae compared to Sham group (Supplementary Figures S2D, E). Additionally, F082, Enterococcaceae and Monoglobaceae were significantly more abundant in the AS-IV group compared to both the Sham and OVX groups (Supplementary Figures S2F, G, I).

At the genus level, the most abundant genera in both the Sham and OVX groups were Muribaculaceae and Prevotellaceae_UCG-003 (Supplementary Figure S3). In the AS_OVX group, Muribaculaceae and *Lactobacillus* were the dominant genera. The OVX and AS_OVX groups were significantly lower than the Sham group in the abundance of Muribaculaceae and Muribaculum, but higher in the abundance of Prevotella and F082. Compared with OVX group, AS-IV treatment significantly increased the abundance of F082 and *Enterococcus*, while decreasing Prevotellaceae_UCG-003. These findings suggest that AS-IV treatment modulates the diversity of gut microbiota in OVX mice.

**FIGURE 7:**
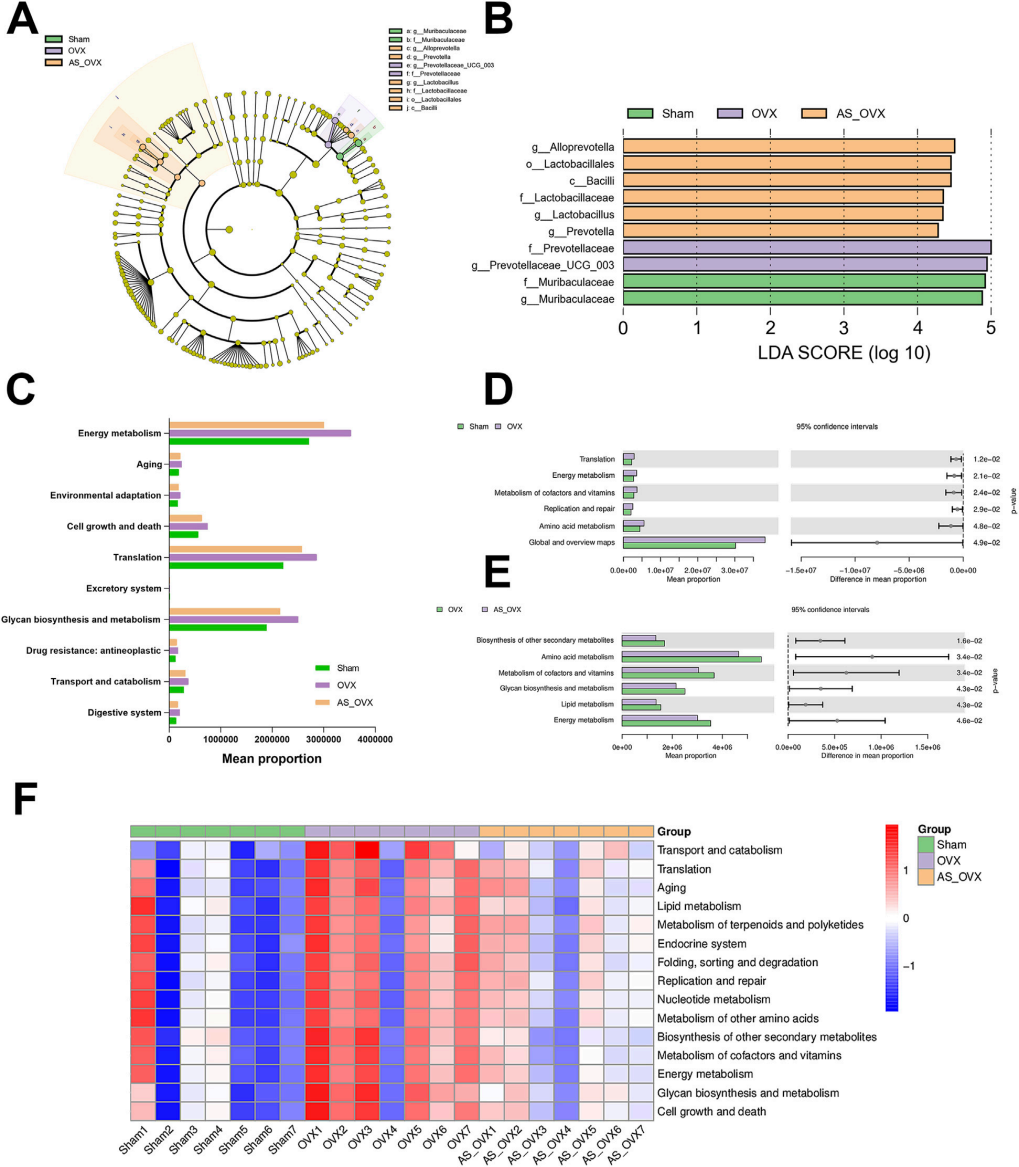
Changes in the composition and function of gut microorganisms. **(A)** Linear discriminant analysis effect size; **(B)** microorganisms with LDA scores greater than four in the three groups; **(C)** KEGG metabolic pathway composition in the gut microorganisms of mice; **(D)** significant differences in the KEGG metabolic pathway between the OVX and Sham groups; **(E)** significant differences in the KEGG metabolic pathway between the AS_OVX and OVX groups; and **(F)** the heatmaps reflect the similarities and differences in the KEGG metabolic pathway different groups.

LEfSe analysis was performed to identify biomarkers among the three groups using Linear Discriminant Analysis (LDA) Effect Size (LEfSe) (Figure 7A). In the Sham group, the enriched bacterial taxa were predominantly from Muribaculaceae. Meanwhile, the OVX group-compromised taxons were Prevotellaceae_UCG_003 and Prevotellaceae (Figure 7B). In the AS_OVX group, enriched bacterial taxa included Alloprovotella, Lactobacillales, Bacilli, Lactobacillaceae, *Lactobacillus* and Prevotella (Figure 7B). Functional predictions of the gut microbiota were conducted using the Kyoto Encyclopedia of Genes and Genomes (KEGG) database (Figure 7C). When comparing the OVX group to the Sham group, significant differences were observed in genes associated with translation, energy metabolism, coenzyme and vitamin metabolism, replication and repair, amino acid metabolism, and global and overview maps (Figure 7D). Comparing the AS_OVX group to the OVX group, significant differences were noted in genes related to the biosynthesis of other secondary metabolites, amino acid metabolism, coenzyme and vitamin metabolism, carbohydrate biosynthesis and metabolism, lipid metabolism, and energy metabolism (Figure 7E). In terms of metabolic pathways, the OVX group showed an upward trend in key pathways such as aging, lipid metabolism, other amino acid metabolism, and energy metabolism. AS-IV treatment in the AS_OVX group mitigated these upregulated trends compared to the OVX group (Figure 7F).

### Association analysis of gut microbiota and bone phenotypes indicators and inflammation cytokines in OVX mice

**FIGURE 8:**
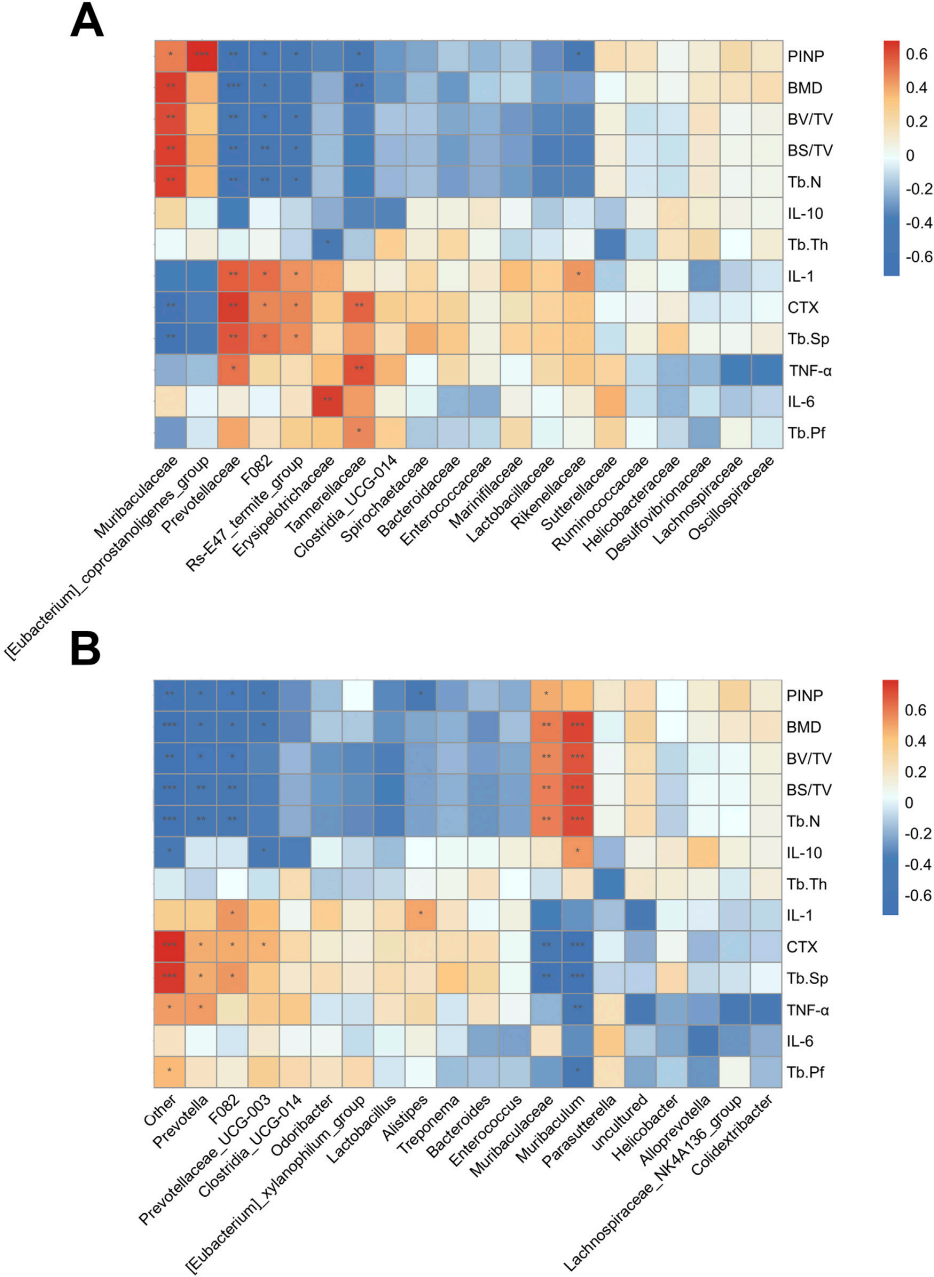
Association analysis of gut microorganisms and bone phenotypes. **(A)** The heat map of the Pearson correlation coefficient between microbe families and bone phenotypes. **(B)** The heat map of the Pearson correlation coefficient between microbe genera and bone phenotypes. *P < 0.05, **P < 0.01 and ***P < 0.001.

At the family level, Muribaculaceae and [Eubacterium]_coprostanoligenes_group were positively correlated with the bone formation markers (BMD, Tb.N, BV/TB, and PINP, etc.). Prevotellaceae, F082, Erysipelotrichaceae, Tannerellaceae, Rs-E47_termite_group and Rikenellaceae were positively correlated with the bone resorption markers (CTX and Tb.Sp) and inflammatory cytokine IL-1, IL-6 and TNF-α (Figure 8A). At the genus level, similar correlations were observed (Figure 8B). These findings suggest that AS-IV treatment may help reduce osteoporosis caused by estrogen deficiency through the regulation of the gut microbiota.

### AS-IV treatment regulated metabolic profiles

**FIGURE 9:**
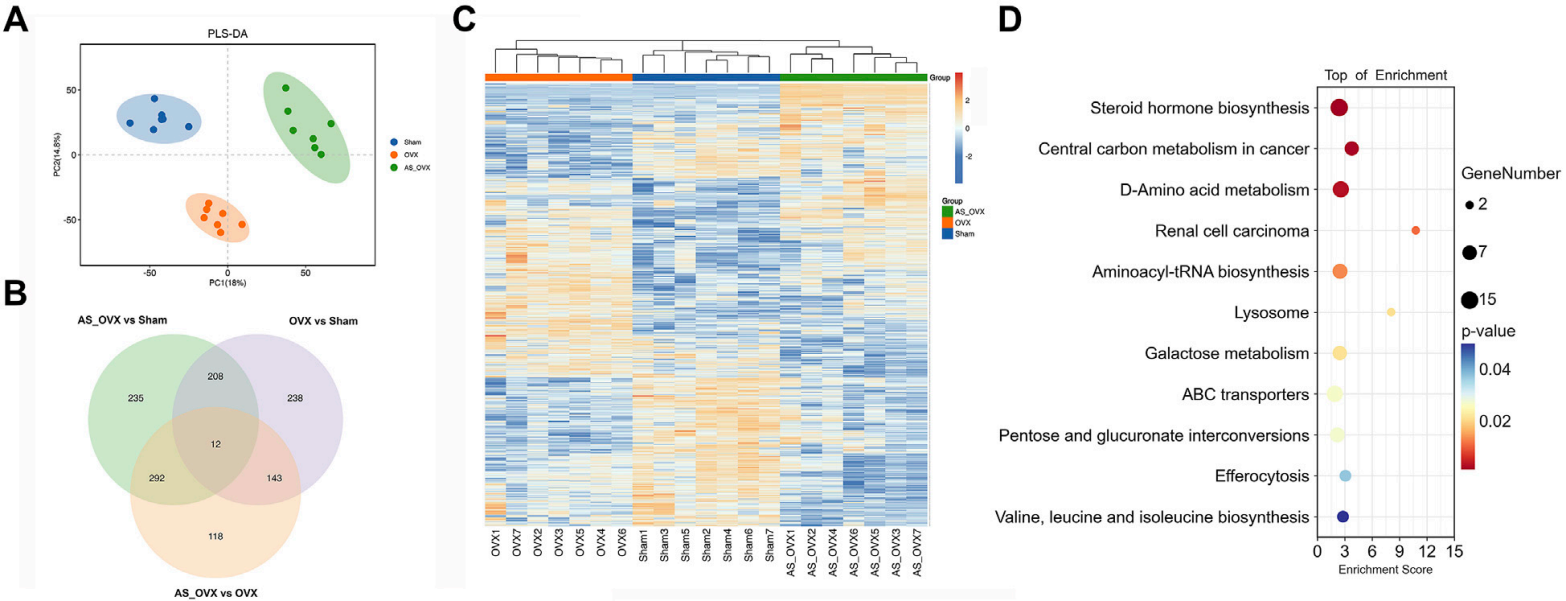
The effects of AS-IV on metabolic pathways. **(A)** Partial least squares discriminant analysis (PLS-DA) of the three groups. **(B)** Venn diagram of the differential metabolites between each other group. **(C)** Heatmap of the differential metabolites. Cluster analysis was carried out by Euclidean Distance. **(D)** The bubble diagram of pathway enrichment analysis of significant metabolites. Node size is based on metabolite number in the pathway. N = 7. Statistical significance was determined by t-test or Mann-Whitney U test.

Principal component analysis (PCA) was performed for each group to evaluate overall metabolic variations, and partial least squares discriminant analysis (PLS-DA) was used for sample classification and validation. Significant differences in metabolism were identified across the groups, with AS-IV treatment inducing distinct metabolic changes in OVX mice (Figure 9A). Differentially expressed metabolites were selected using a p-value threshold <0.05 and an absolute fold change (FC) >4. A total of 601 metabolites were differentially expressed between the OVX and Sham groups, 565 between the AS_OVX and OVX groups, and 747 between the AS_OVX and Sham groups (Figure 9B). A hierarchical clustering heatmap was generated to visualize these metabolic changes, revealing significant metabolic differences among the three groups (Figure 9C). These findings indicate that AS-IV treatment significantly alters the metabolic profile in OVX mice.

Differentially expressed metabolites were visualized using volcano plots (Supplementary Figures S4A–C). The amounts of the Carbacyclin, PG(PGE1/a-17:0), Thiamine, 11-deoxy-11-methylene-15-keto-PGD2 and Drostanolone were decrease in OVX group, while Rociletinib, Val His Ile, Melagatran, 19-hydroxybufalin-3-(16-hydroxy-palmitate) and 9S-HpHtrE were increased, compared to Sham group. The amounts of the Rociletinib, 4-Ethylphenol glucuronide, Val His Ile, Melagatran, and Indoxyl sulfate were decrease in AS_OVX group, while Enkephalin L, 17-Dimethylaminogeldanamycin, Ganoderic acid G, Astragaloside A and B-Octylglucoside were increased, compared to OVX group.

Differentially expressed metabolites analyzed using the KEGG database to explore their functions. A total of 11 metabolic pathways were significantly regulated (p < 0.05), including steroid hormone biosynthesis, D-amino acid metabolism, aminoacyl-tRNA biosynthesis, lysosome, galactose metabolism, ABC transporters, pentose and glucuronate interconversions, and valine, leucine, and isoleucine biosynthesis. The pathway regulation is visualized in Figure 9D, with additional KEGG analysis between OVX and Sham group, AS_OVX and OVX group were shown in Supplementary Figure S5.

### Association analysis of gut microbiota, metabolites, and bone phenotypes, serum inflammatory cytokines

**FIGURE 10:**
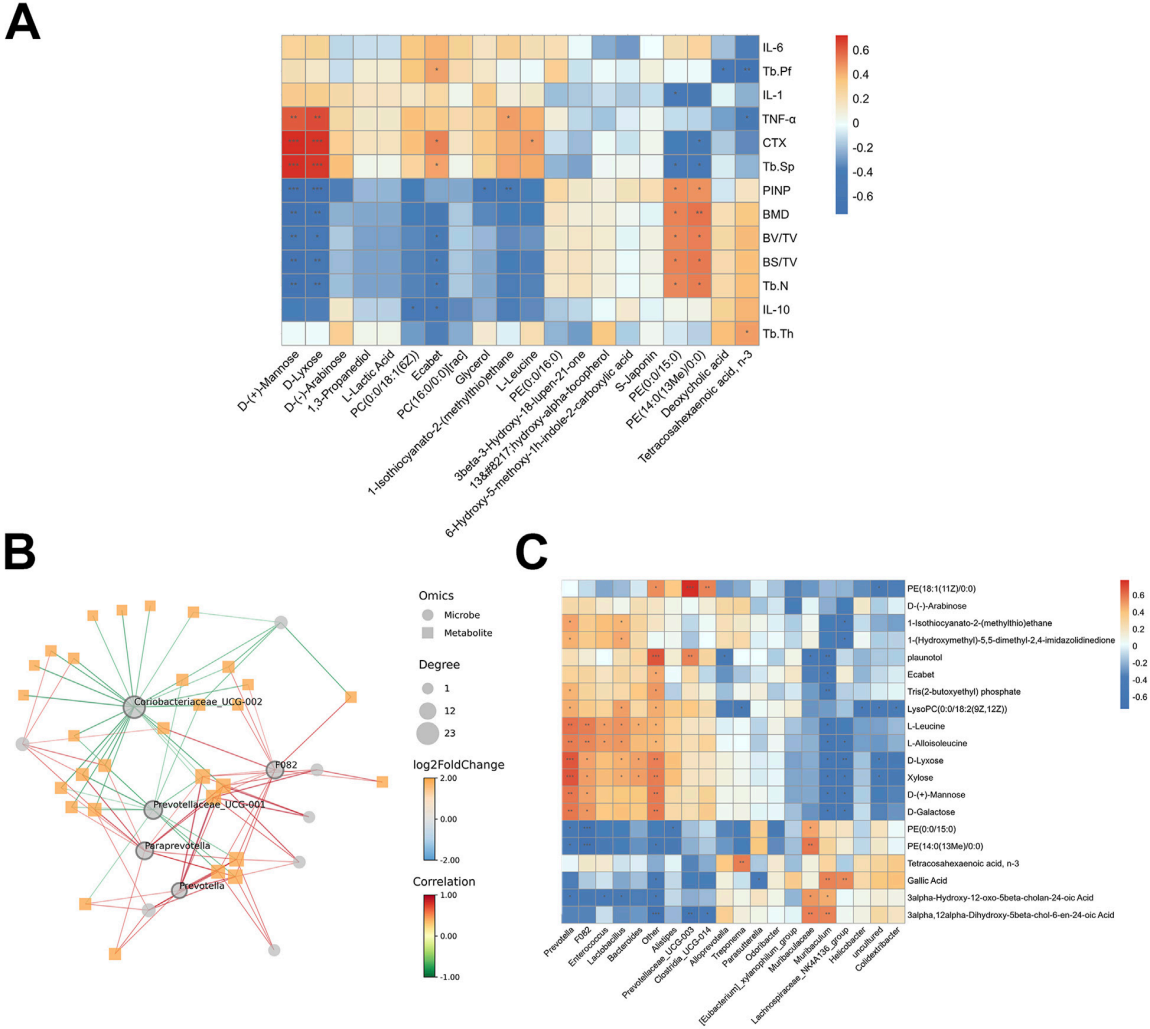
The relationship between gut microbiota, metabolites and bone phenotypes, serum inflammatory cytokines. **(A)** The heat map of the Pearson correlation coefficient between gut metabolites and bone phenotypes, serum inflammatory cytokines. **(B)** Network diagram between bacteria and metabolites. **(C)** The heat map of the Pearson correlation coefficient between bacteria and metabolites. *p < 0.05, **p < 0.01, ***p < 0.001.

Correlation analysis of differential metabolites with bone phenotypes and serum inflammatory cytokines is shown in Figure 10A. Metabolites positively correlated with bone formation markers (e.g., BMD, Tb.N, BV/TV, and PINP) included PE (0:0/15:0) and PE [14:0(13Me)/0:0]. Conversely, metabolites such as D-Mannose, D-Lyxose, and Ecabet were positively correlated with bone resorption markers (CTX, Tb.Sp) and pro-inflammatory cytokine TNF-α.

Correlation analysis revealed a strong association between specific bacteria and key metabolites (Figures 10B, C). Prevotellaceae_UCG_001, F082, Prevotella, Paraprevotella and Coriobateriaceae_UCG_002 had the highest centrality scores (degree of node connectivity) (Figure 10B). Prevotella and F082 were positively correlated with D-Lyxose, Xylose, L-Leucine and L-Alloisoleucine, but were negatively correlated with PE (0:0/15:0) and PE [14:0(13Me)/0:0]. Muribaculum and Lachnospiraceae_NK4A136_group were negatively correlated with D-Lyxose, Xylose, and L-Alloisoleucine, but were positively correlated with Gallic acid (Figure 10C). These metabolites may represent key targets through which AS-IV exerts its beneficial effects on osteoporosis in ovariectomized rats.

## Discussion

AS-IV treatment effectively mitigated bone loss after OVX, via increasing bone formation and decreasing bone resorption. AS-IV treatment strengthened the intestinal barrier function and decreased gut permeability. AS-IV alleviated oxidative stress in colon and lowered serum levels of IL-1, IL-6 and INF-α in serum. In addition, AS-IV modifies the composition of gut microbiota. Specifically, AS-IV induced significant changes in the gut microbiota, increasing the abundance of Alloprovotella, Lactobacillales, Bacilli, Lactobacillaceae, *Lactobacillus* and Prevotella, simultaneously downregulates that of Tannerellaceae and Prevotellaceae_UCG-003. Furthermore, AS-IV altered the gut metabolic profile, affecting amino acids and phosphatidylethanolamine (PE) derivatives. These metabolic changes were closely associated with the abundance of bacteria such as Prevotellaceae_UCG_001, F082, Prevotella, Muribaculum and Lachnospiraceae_NK4A136_group etc.

The intestinal mucosal barrier is crucial for regulating systemic inflammatory responses. Previous studies have shown that the ovariectomy (OVX) model impairs gut barrier function and is strongly associated with inflammation (Guo et al., 2023; Zhang et al., 2022). AS-IV was indicated to promote intestinal barrier function (Li et al., 2023). Tight junction proteins (TJs) such as ZO-1, claudin-1, and occludin are key to maintaining intestinal permeability (Zhang et al., 2022). In this study, AS-IV treatment significantly improved the expression of these TJs and alleviated gut barrier damage compared to the OVX group, highlighting its protective role in intestinal function.

PMOP is closely associated with oxidative damage and systemic chronic inflammation. Estrogen deficiency leads to excessive accumulation of reactive oxygen species (ROS) and free radicals, which activate inflammatory pathways. AS-IV has demonstrated anti-inflammatory and antioxidant properties (Zhou et al., 2020), mitigating OVX-induced oxidative stress by enhancing the levels of SOD) and glutathione (GSH) while reducing ROS levels. PMOP is characterized by a chronic inflammatory state, with increased levels of pro-inflammatory cytokines such as IL-1β, IL-6, and TNF-α, along with reduced levels of anti-inflammatory cytokines like IL-10 (Li et al., 2020). ELISA analysis in this study revealed that compared to the Sham group, the OVX group exhibited significantly elevated levels of IL-1β, IL-6, and TNF-α in both serum and bone marrow, while IL-10 levels were significantly reduced. AS-IV treatment effectively alleviated the inflammation induced by OVX by lowering IL-1β, IL-6, and TNF-α levels and increasing IL-10 levels. These findings suggest that AS-IV not only reduces oxidative stress but also suppresses the inflammatory response in OVX mice, thereby attenuating bone loss. The dual antioxidant and anti-inflammatory effects of AS-IV highlight its therapeutic potential in treating PMOP by targeting both oxidative damage and chronic inflammation, which are key factors in the pathogenesis of osteoporosis.

In the OVX-induced animal models, significant gut microbiota dysbiosis was observed after surgery (Lan et al., 2022; Lu et al., 2021). The gut-bone axis has been reported in previous studies to play a crucial role in the pathophysiology of osteoporosis. Estrogen deficiency can significantly alter the abundance of various microbial species, contributing to bone loss (Wang et al., 2021b; Zhang et al., 2022). Thus, restoring gut homeostasis has emerged as a novel therapeutic target for osteoporosis (D’Amelio and Sassi, 2018). According to our results, AS-IV treatment increased the abundances of *Lactobacillus*, Allopreotella, Prevotella and Enterooccus. Numerous studies have reported the beneficial effects of *Lactobacillus* on bone homeostasis highlighting its potential mechanisms of action. Similarly, Prevotella has been demonstrated to improve bone loss in OVX mice by optimizing gut permeability and inhibiting the release of osteoclast-promoting factors. Guo et al. reported that *Lactobacillus* alleviated osteoporosis caused by estrogen deficiency by modulating the gut microbiome and strengthening the intestinal barrier, while also promoting the balance between Th17 and Treg cells in both the gut and bone (Guo et al., 2023). Sapra et al. demonstrated that *Lactobacillus* rhamnosus reduced bone degradation and preserved bone integrity by modulating the balance between Treg and Th17 cells in ovariectomized mice (Sapra et al., 2021). Prevotella was found to mitigate bone loss in ovariectomized mice by enhancing intestinal barrier function and suppressing the release of cytokines that promote osteoclastogenesis (Zhang et al., 2023b; Wang et al., 2021b).

Duan et al. demonstrated that Prevotella alleviated cognitive impairment in vascular dementia rats by lowering oxidative stress and reducing the release of pro-inflammatory cytokines (Duan et al., 2023). All of these results suggest that the modulating gut microbiota, with increased the abundance of probiotics (*Lactobacillus* and Prevotella), may play a key role in the therapeutic effects of AS-IV in OVX-induced osteoporosis.

Microbiota-related metabolites act as key mediators between the gut and bone during the process of OVX-induced bone loss. AS-IV significantly alters the production of microbiota-related amino acids and linoleic acid, which play important roles in bone metabolism (Du et al., 2022). Fecal metabolomics revealed that AS-IV treatment markedly changed the metabolic profiles in OVX mice. The different fecal metabolites were enriched in pathways such as steroid hormone biosynthesis, D-amino acid metabolism, galactose metabolism, pentose and glucuronate transformations, as well as the biosynthesis of valine, leucine, and isoleucine. D-Mannose helps to mitigate bone loss in weightless conditions by inhibiting the fusion of osteoclast cells (Gu et al., 2023). Additionally, D-mannose exhibits anti-inflammatory properties, suppressing neutrophil oxidative bursts and oxidative reactions in experimental neuroinflammation (Rest et al., 1988; Wang et al., 2021a). D-mannose was reported to suppress macrophage IL-1B production via the suppression of succinatemediated HIF-1a activation (Torretta et al., 2020). The increased levels of PEs were found in the low bone mineral density patients (Aleidi et al., 2022). Phosphatidylethanolamine (PE), essential components of cell membranes, play critical roles in cellular fusion, mitochondrial stability, and autophagy (Irie et al., 2017; Verma et al., 2018). Some studies *in vitro* revealed that the intracellular concentration of PEs is linked to osteoclastogenesis, and their levels increase during osteoclast differentiation (Kitano et al., 2020; Irie et al., 2017). Moreover, several metabolites significantly associated with inflammation and bone factors showed strong correlations with the abundance of specific bacterial genera, which was consistent with previous studies. The accumulation of D-galactose derived from the gut microbiota and intestinal dysbiosis is associated with cellular senescence (Gao et al., 2023). Chronic hypoxia leads to a striking reduction in the amount of *Lactobacillus* and D-galactosedegrading enzymes in the gut, producing excess reactive oxygen species (ROS) and inducing senescence in bone marrow mesenchymal stem cells (Xing et al., 2018). The accumulation of D-galactose can subsequently affect the gut microbiota. For example, D-galactose-induced aging model downregulates the relative abundance of Muribaculaceae (Xu et al., 2024). Gallic acid (GA), 3,4,5-trihydroxybenzoic acid, is a polyphenol compound and has various kinds of biological and pharmacological activities, including antioxidant, antimicrobial, anticancer, anti-inflammatory, and metabolic disease prevention activities (Yang et al., 2020). The effect between the gut microbiota and GA is mutual; intestinal bacteria has the ability to metabolize GA, and GA also can induce changes in the microbiota toward a more favorable composition and activity, including the production of short-chain fatty acids (SCFAs) in the colon (Yang et al., 2020; Pandurangan et al., 2015). These metabolites could represent key targets through which AS-IV exerts its therapeutic effects on osteoporosis. Further studies are needed to explore how these metabolites influence bone health and their interaction with the gut microbiota, which may provide new insights into the treatment of osteoporosis.

This study has several limitations. First, the composition of the gut microbiota is influenced by factors such as diet and ethnicity, which can be controlled in rodent experiments but pose significant challenges in human studies. Second, given the dynamic nature of gut microbiota, longitudinal studies are needed to fully understand the long-term effects of AS-IV on gut microbiota and bone health. Third, the 16S rRNA sequencing technique, which was utilized to analyze the distribution, classification, and diversity of GM in OP diseases, still lacks the ability to differentiate between active and non-active microbiomes, and there is a notable absence of resolution beyond the genus level. Forth, although certain key metabolites (e.g., D-mannose and phosphatidylethanolamine) are suggested to be associated with bone metabolism, further research is required to investigate the detailed mechanisms by which AS-IV modulates gut microbiota and metabolites, and how these changes influence bone health. Fifth, while the results in mice are promising, further clinical trials are necessary to confirm the efficacy and safety of AS-IV in human subjects. While this research provides a preliminary assessment of the potential mechanisms by which AS-IV exerts its effects via the gut microbiota, further studies are necessary to fully elucidate the specific relationship between AS-IV’s anti-osteoporotic effects and the gut microbiome.

## Conclusion

The findings of this study demonstrate that AS-IV exerts anti-osteoporotic effects in an OVX-induced mouse model of osteoporosis. This effect is likely closely linked to AS-IV’s impact on gut microbiota composition and metabolism. These results provide new insights into AS-IV as a potential therapeutic for osteoporosis and highlight its influence on gut microbiota and serum metabolism.

## Data Availability

The datasets presented in this study can be found in online repositories. The repository name and accession number of 16S rDNA sequencing data can be found below: https://bigd.big.ac.cn/gsa/browse/CRA023955, BioProject accession: PRJCA037525. The repository name and accession number of metabolomic profiling data can be found below: https://ngdc.cncb.ac.cn/omix/releaseList, BioProject accession: PRJCA037593.

## Abbreviations

AS-IV, astragaloside IV; PMOP, postmenopausal osteoporosis; OVX, ovariectomy; POP, primary osteoporosis; IL-1, interleukin-1; IL-6, interleukin-6; TNF-α, tumor necrosis factor-alpha; ROS, reactive oxygen species; LPS, lipopolysaccharide; Micro-CT, micro-computed tomography, BMD, bone mineral density; BV/TV, bone volume fraction; BS/TV, bone surface area to total volume ratio; Tb.N, trabecular number; Tb.Th, trabecular thickness; Tb.Sp, trabecular separation; GSH, reduced glutathione; SOD, superoxide dismutase; AB-PAS, Alcian Blue-Periodic Acid Schiff; TRAP, tartrate-resistant acid phosphatase; OCN, osteocalcin; PINP, procollagen type I N-terminal propeptide; CTX-I, C-terminal telopeptide of type I collagen; ELISA, enzyme-linked immunosorbent assay; GO, Gene Ontology; KEGG, Kyoto Encyclopedia of Genes and Genome; WB, Western Blot; GC-MS, gas chromatography-mass spectrometry; PLS-DA, partial least-squares discriminant analysis.

## References

[B1] Aleidi S. M. Al-Ansari M. M. Alnehmi E. A. Malkawi A. K. Alodaib A. Alshaker M. (2022). Lipidomics profiling of patients with low bone mineral density (LBMD). Int. J. Mol. Sci. 23, 12017. 10.3390/ijms231912017 36233318 PMC9570421

[B2] Almeida M. Han L. Martin-Millan M. Plotkin L. I. Stewart S. A. Roberson P. K. (2007). Skeletal involution by age-associated oxidative stress and its acceleration by loss of sex steroids. J. Biol. Chem. 282, 27285–27297. 10.1074/jbc.M702810200 17623659 PMC3119455

[B3] Bian Q. Huang J. H. Liang Q. Q. Shu B. Hou W. Xu H. (2011). The osteogenetic effect of astragaloside IV with centrifugating pressure on the OCT-1 cells. Pharmazie 66, 63–68. 21391437

[B4] Bonaccorsi G. Piva I. Greco P. Cervellati C. (2018). Oxidative stress as a possible pathogenic cofactor of post-menopausal osteoporosis: existing evidence in support of the axis oestrogen deficiency-redox imbalance-bone loss. Indian J. Med. Res. 147, 341–351. 10.4103/ijmr.IJMR_524_18 29998869 PMC6057254

[B5] Boyd S. K. Davison P. Muller R. Gasser J. A. (2006). Monitoring individual morphological changes over time in ovariectomized rats by *in vivo* micro-computed tomography. Bone 39, 854–862. 10.1016/j.bone.2006.04.017 16757220

[B6] Chen J. K. Guo M. K. Bai X. H. Chen L. Q. Su S. M. Li L. (2020). Astragaloside IV ameliorates intermittent hypoxia-induced inflammatory dysfunction by suppressing MAPK/NF-κB signalling pathways in Beas-2B cells. Sleep. Breath. 24, 1237–1245. 10.1007/s11325-019-01947-8 31907823

[B7] Cheng C. Liu K. Shen F. Zhang J. Xie Y. Li S. (2023). Astragaloside IV targets PRDX6, inhibits the activation of RAC subunit in NADPH oxidase 2 for oxidative damage. Phytomedicine 114, 154795. 10.1016/j.phymed.2023.154795 37030053

[B8] D'Amelio P. Sassi F. (2018). Gut microbiota, immune system, and bone. Calcif. Tissue Int. 102, 415–425. 10.1007/s00223-017-0331-y 28965190

[B9] Du X. Q. Shi L. P. Chen Z. W. Hu J. Y. Zuo B. Xiong Y. (2022). Astragaloside IV ameliorates isoprenaline-induced cardiac fibrosis in mice via modulating gut microbiota and fecal metabolites. Front. Cell Infect. Microbiol. 12, 836150. 10.3389/fcimb.2022.836150 35656031 PMC9152365

[B10] Duan R. Hou J. Wang X. Huang Z. Cao H. Hu J. (2023). Prevotella histicola transplantation ameliorates cognitive impairment and decreases oxidative stress in vascular dementia rats. Brain Sci. 13, 1136. 10.3390/brainsci13081136 37626492 PMC10452631

[B11] Gao H. Nepovimova E. Heger Z. Valko M. Wu Q. Kuca K. (2023). Role of hypoxia in cellular senescence. Pharmacol. Res. 194, 106841. 10.1016/j.phrs.2023.106841 37385572

[B12] Gray A. I. Igoli J. O. Edrada-Ebel R. (2012). Natural products isolation in modern drug discovery programs. Methods Mol. Biol. 864, 515–534. 10.1007/978-1-61779-624-1_20 22367910

[B13] Guo M. Liu H. Yu Y. Zhu X. Xie H. Wei C. (2023). Lactobacillus rhamnosus GG ameliorates osteoporosis in ovariectomized rats by regulating the Th17/Treg balance and gut microbiota structure. Gut Microbes 15, 2190304. 10.1080/19490976.2023.2190304 36941563 PMC10038048

[B14] Gu R. Liu H. Hu M. Zhu Y. Liu X. Wang F. (2023). D-Mannose prevents bone loss under weightlessness. J. Transl. Med. 21, 8. 10.1186/s12967-022-03870-1 36617569 PMC9827691

[B15] Hansen M. S. Frost M. (2022). Alliances of the gut and bone axis. Semin. Cell Dev. Biol. 123, 74–81. 10.1016/j.semcdb.2021.06.024 34303607

[B16] Irie A. Yamamoto K. Miki Y. Murakami M. (2017). Phosphatidylethanolamine dynamics are required for osteoclast fusion. Sci. Rep. 7, 46715. 10.1038/srep46715 28436434 PMC5402267

[B17] Kitano V. J. F. Ohyama Y. Hayashida C. Ito J. Okayasu M. Sato T. (2020). LDL uptake-dependent phosphatidylethanolamine translocation to the cell surface promotes fusion of osteoclast-like cells. J. Cell Sci. 133, jcs243840. 10.1242/jcs.243840 32295848

[B18] Lan H. Liu W. H. Zheng H. Feng H. Zhao W. Hung W. L. (2022). Bifidobacterium lactis BL-99 protects mice with osteoporosis caused by colitis via gut inflammation and gut microbiota regulation. Food Funct. 13, 1482–1494. 10.1039/d1fo02218k 35060590

[B19] Leboff M. S. Greenspan S. L. Insogna K. L. Lewiecki E. M. Saag K. G. Singer A. J. (2022). The clinician's guide to prevention and treatment of osteoporosis. Osteoporos. Int. 33, 2049–2102. 10.1007/s00198-021-05900-y 35478046 PMC9546973

[B20] Li J. Chen X. Lu L. Yu X. (2020). The relationship between bone marrow adipose tissue and bone metabolism in postmenopausal osteoporosis. Cytokine Growth Factor Rev. 52, 88–98. 10.1016/j.cytogfr.2020.02.003 32081538

[B21] Li L. Chen B. Zhu R. Li R. Tian Y. Liu C. (2019). Fructus Ligustri Lucidi preserves bone quality through the regulation of gut microbiota diversity, oxidative stress, TMAO and Sirt6 levels in aging mice. Aging (Albany NY) 11, 9348–9368. 10.18632/aging.102376 31715585 PMC6874471

[B22] Li M. Wang W. Geng L. Qin Y. Dong W. Zhang X. (2015). Inhibition of RANKL-induced osteoclastogenesis through the suppression of the ERK signaling pathway by astragaloside IV and attenuation of titanium-particle-induced osteolysis. Int. J. Mol. Med. 36, 1335–1344. 10.3892/ijmm.2015.2330 26324422

[B23] Li Z. Hu E. Zheng F. Wang S. Zhang W. Luo J. (2023). The effects of astragaloside IV on gut microbiota and serum metabolism in a mice model of intracerebral hemorrhage. Phytomedicine 121, 155086. 10.1016/j.phymed.2023.155086 37783132

[B24] Lu L. Chen X. Liu Y. Yu X. (2021). Gut microbiota and bone metabolism. Faseb J. 35, e21740. 10.1096/fj.202100451R 34143911

[B25] Pandurangan A. K. Mohebali N. Esa N. M. Looi C. Y. Ismail S. Saadatdoust Z. (2015). Gallic acid suppresses inflammation in dextran sodium sulfate-induced colitis in mice: possible mechanisms. Int. Immunopharmacol. 28, 1034–1043. 10.1016/j.intimp.2015.08.019 26319951

[B26] Reid I. R. Billington E. O. (2022). Drug therapy for osteoporosis in older adults. Lancet 399, 1080–1092. 10.1016/S0140-6736(21)02646-5 35279261

[B27] Rest R. F. Farrell C. F. Naids F. L. (1988). Mannose inhibits the human neutrophil oxidative burst. J. Leukoc. Biol. 43, 158–164. 10.1002/jlb.43.2.158 2826631

[B28] Rosen C. J. (2000). “The epidemiology and pathogenesis of osteoporosis,” in Endotext. Editors Feingold k. R. Anawalt B. Boyce A. Chrousos G. De herder W. W. Dungan K. (South Dartmouth (MA): MDText.com, Inc.).

[B29] Sapra L. Dar H. Y. Bhardwaj A. Pandey A. Kumari S. Azam Z. (2021). Lactobacillus rhamnosus attenuates bone loss and maintains bone health by skewing Treg-Th17 cell balance in Ovx mice. Sci. Rep. 11, 1807. 10.1038/s41598-020-80536-2 33469043 PMC7815799

[B30] Shi Y. H. Zhang X. L. Ying P. J. Wu Z. Q. Lin L. L. Chen W. (2021). Neuroprotective effect of astragaloside IV on cerebral ischemia/reperfusion injury rats through sirt1/mapt pathway. Front. Pharmacol. 12, 639898. 10.3389/fphar.2021.639898 33841157 PMC8033022

[B31] Soares H. D. (2010). The use of mechanistic biomarkers for evaluating investigational CNS compounds in early drug development. Curr. Opin. Investig. Drugs 11, 795–801. 20571975

[B32] Tanabe K. Nakamura S. Moriyama-Hashiguchi M. Kitajima M. Ejima H. Imori C. (2019). Dietary fructooligosaccharide and glucomannan alter gut microbiota and improve bone metabolism in senescence-accelerated mouse. J. Agric. Food Chem. 67, 867–874. 10.1021/acs.jafc.8b05164 30632742

[B33] Torretta S. Scagliola A. Ricci L. Mainini F. Di Marco S. Cuccovillo I. (2020). D-mannose suppresses macrophage IL-1β production. Nat. Commun. 11, 6343. 10.1038/s41467-020-20164-6 33311467 PMC7733482

[B34] Verma S. K. Leikina E. Melikov K. Gebert C. Kram V. Young M. F. (2018). Cell-surface phosphatidylserine regulates osteoclast precursor fusion. J. Biol. Chem. 293, 254–270. 10.1074/jbc.M117.809681 29101233 PMC5766907

[B35] Wang J. Jalali Motlagh N. Wang C. Wojtkiewicz G. R. Schmidt S. Chau C. (2021a). d-mannose suppresses oxidative response and blocks phagocytosis in experimental neuroinflammation. Proc. Natl. Acad. Sci. U. S. A. 118, e2107663118. 10.1073/pnas.2107663118 34702739 PMC8673064

[B36] Wang Z. Chen K. Wu C. Chen J. Pan H. Liu Y. (2021b). An emerging role of Prevotella histicola on estrogen deficiency-induced bone loss through the gut microbiota-bone axis in postmenopausal women and in ovariectomized mice. Am. J. Clin. Nutr. 114, 1304–1313. 10.1093/ajcn/nqab194 34113963

[B37] Wu S. Chen Z. (2019). Astragaloside IV alleviates the symptoms of experimental ulcerative colitis *in vitro* and *in vivo* . Exp. Ther. Med. 18, 2877–2884. 10.3892/etm.2019.7907 31572532 PMC6755457

[B38] Xing J. Ying Y. Mao C. Liu Y. Wang T. Zhao Q. (2018). Hypoxia induces senescence of bone marrow mesenchymal stem cells via altered gut microbiota. Nat. Commun. 9, 2020. 10.1038/s41467-018-04453-9 29789585 PMC5964076

[B39] Xu Y. Xue M. Li J. Ma Y. Wang Y. Zhang H. (2024). Fucoidan improves D-galactose-induced cognitive dysfunction by promoting mitochondrial biogenesis and maintaining gut microbiome homeostasis. Nutrients 16, 1512. 10.3390/nu16101512 38794753 PMC11124141

[B40] Yang K. Zhang L. Liao P. Xiao Z. Zhang F. Sindaye D. (2020). Impact of gallic acid on gut health: focus on the gut microbiome, immune response, and mechanisms of action. Front. Immunol. 11, 580208. 10.3389/fimmu.2020.580208 33042163 PMC7525003

[B41] Zhang Y. W. Cao M. M. Li Y. J. Lu P. P. Dai G. C. Zhang M. (2022). Fecal microbiota transplantation ameliorates bone loss in mice with ovariectomy-induced osteoporosis via modulating gut microbiota and metabolic function. J. Orthop. Transl. 37, 46–60. 10.1016/j.jot.2022.08.003 PMC9520092 36196151

[B42] Zhang Y. W. Cao M. M. Li Y. J. Sheng R. W. Zhang R. L. Wu M. T. (2023b). The preventive effects of probiotic Prevotella histicola on the bone loss of mice with ovariectomy-mediated osteoporosis. Microorganisms 11, 950. 10.3390/microorganisms11040950 37110373 PMC10146713

[B43] Zhang C. Li H. Li J. Hu J. Yang K. Tao L. (2023a). Oxidative stress: a common pathological state in a high-risk population for osteoporosis. Biomed. Pharmacother. 163, 114834. 10.1016/j.biopha.2023.114834 37163779

[B44] Zhang J. Wu C. Gao L. Du G. Qin X. (2020). Astragaloside IV derived from Astragalus membranaceus: a research review on the pharmacological effects. Adv. Pharmacol. 87, 89–112. 10.1016/bs.apha.2019.08.002 32089240

[B45] Zhong Y. Liu W. Xiong Y. Li Y. Wan Q. Zhou W. (2022). Astragaloside Ⅳ alleviates ulcerative colitis by regulating the balance of Th17/Treg cells. Phytomedicine 104, 154287. 10.1016/j.phymed.2022.154287 35752072

[B46] Zhou L. Zhang R. Yang S. Zhang Y. Shi D. (2020). Astragaloside IV alleviates placental oxidative stress and inflammation in GDM mice. Endocr. Connect. 9, 939–945. 10.1530/EC-20-0295 33006955 PMC7583135

[B47] Zhu S. He H. Gao C. Luo G. Xie Y. Wang H. (2018). Ovariectomy-induced bone loss in TNFα and IL6 gene knockout mice is regulated by different mechanisms. J. Mol. Endocrinol. 60, 185–198. 10.1530/JME-17-0218 29339399

