# Supplementary material for: Astragaloside IV alleviated bone loss in mice with ovariectomy-induced osteoporosis via modulating gut microbiota and fecal metabolism
Source: Front Pharmacol. 2025 Apr 3;16:1548491. doi: 10.3389/fphar.2025.1548491 (PMC12003300; doi:10.3389/fphar.2025.1548491)
Supplement: Supplementary file 1 [file DataSheet1.docx]

Supplementary materials

**Supplementary Table 1** The scoring criteria of HE staining of colon

|  | Crypt damage | Goblet cells loss | Inflammatory cell infiltration |
| --- | --- | --- | --- |
| 0 | none | none | none |
| 1 | 0-10% | mild | mild |
| 2 | 10-20% | moderate | moderate |
| 3 | 10-20% | severe | severe |

**
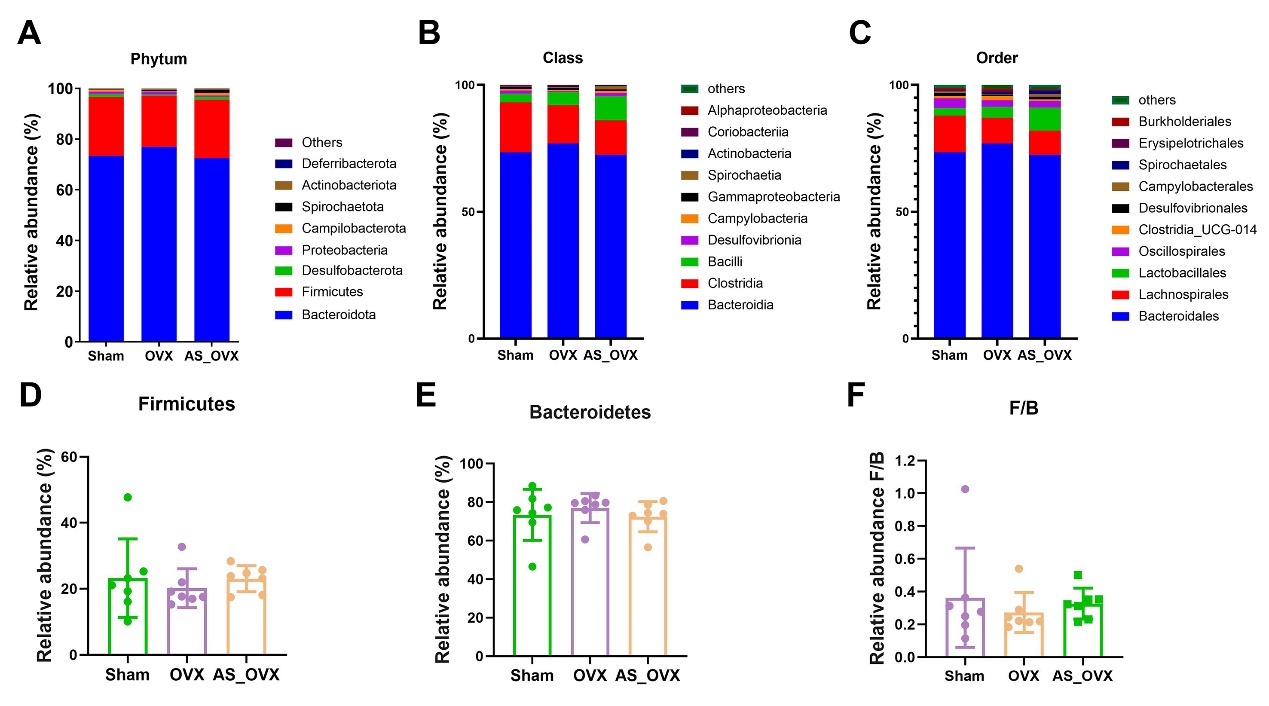
Supplementary Figure 1. Impact of AS-IV on the microflora of mice at the phylum, class and order levels.** Abundance of the intestinal microbe phyla (A), intestinal microbe classes (B), intestinal microbe orders (C), Firmicutes (D), Bacteroidota (E) and F/B (F). F/B, ratio of Firmicutes to Bacteroidota. Data are mean ± SD (n=7).

**
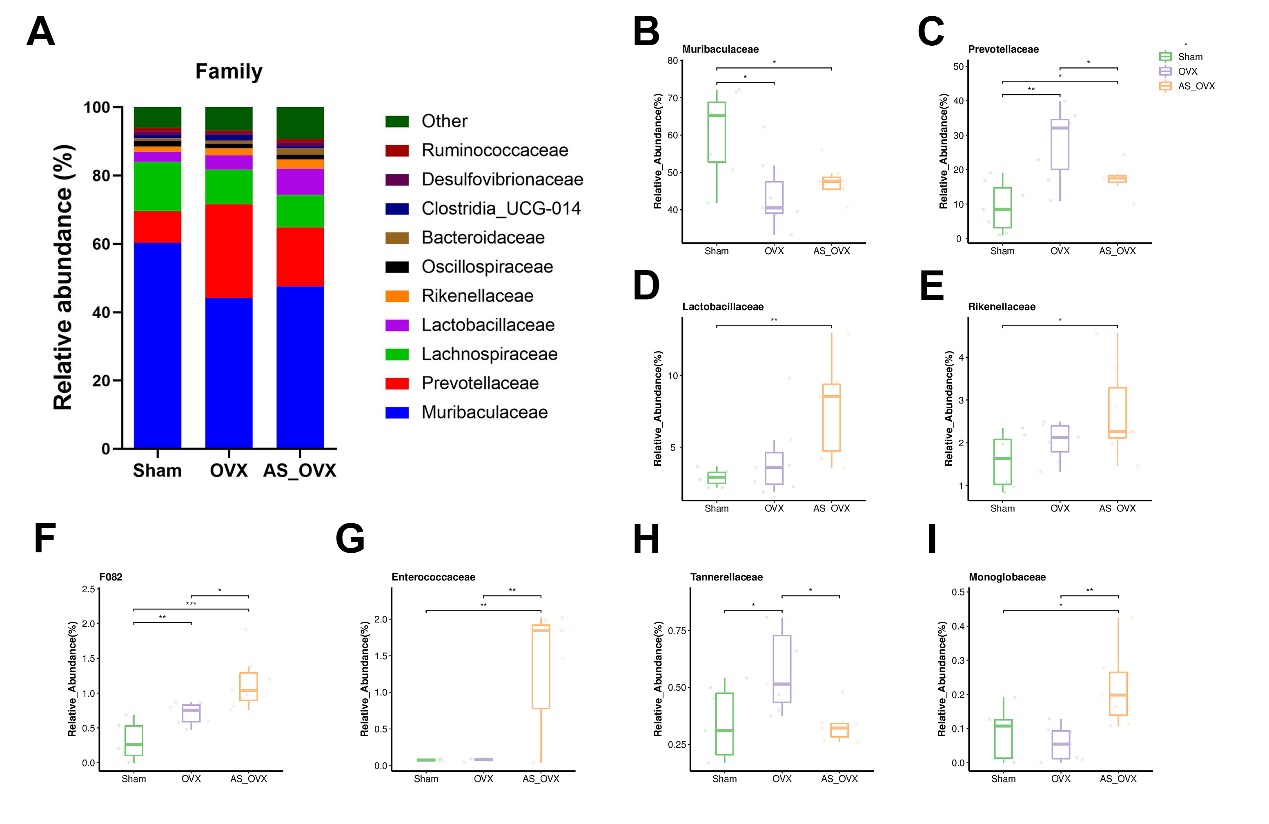
Supplementary Figure 2. Impact of AS-IV on the microflora of mice at the family level.** Abundance of the intestinal microbe families (A), Muribaculaceae (B), Prevotellaceae (C), Lactobacillaceae (D), Rikenellaceae (E), F082 (F), Enterococcaceae (G), Tannerellaceae (H), Monoglobaceae (I). Data are mean ± SD (n = 7).

**
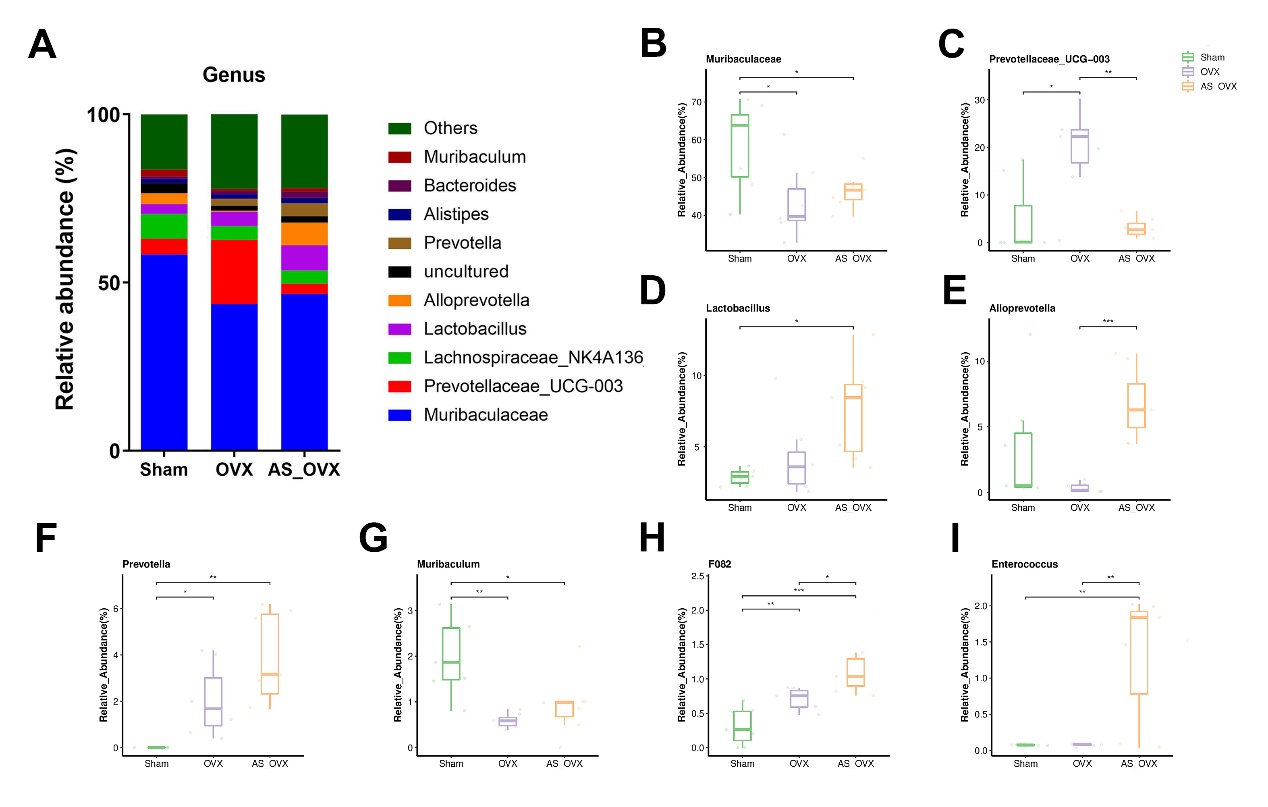
Supplementary Figure 3. Impact of AS-IV on the microflora of mice at the genus level.** Abundance of the intestinal microbe genera (A), Muribaculaceae (B), Prevotellaceae_UCG_003 (C), Lactobacillus (D), Alloprevotella (E), Prevotella (F), Muribaculum (G), F082 (H), Enterococcus (I). Data are mean ± SD (n = 7).


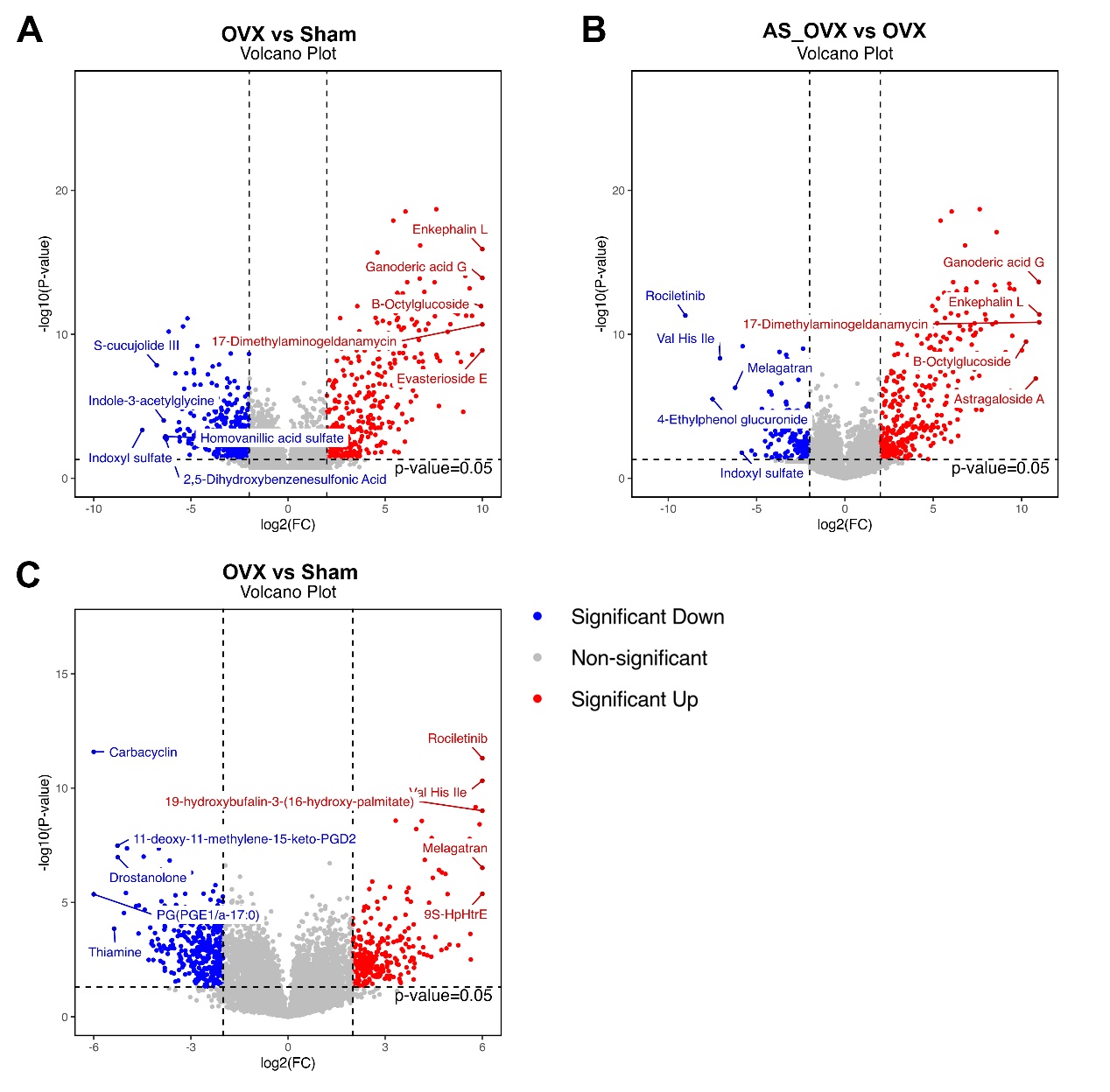


**Supplementary Figure 4. Metabolomics profiles of mice in Sham, OVX and AS_OVX groups.** (A) Volcano plot of differential metabolites between OVX and Sham group. (B) Volcano plot of differential metabolites between AS_OVX and OVX group. (C) Volcano plot of differential metabolites between AS_OVX and Sham group.


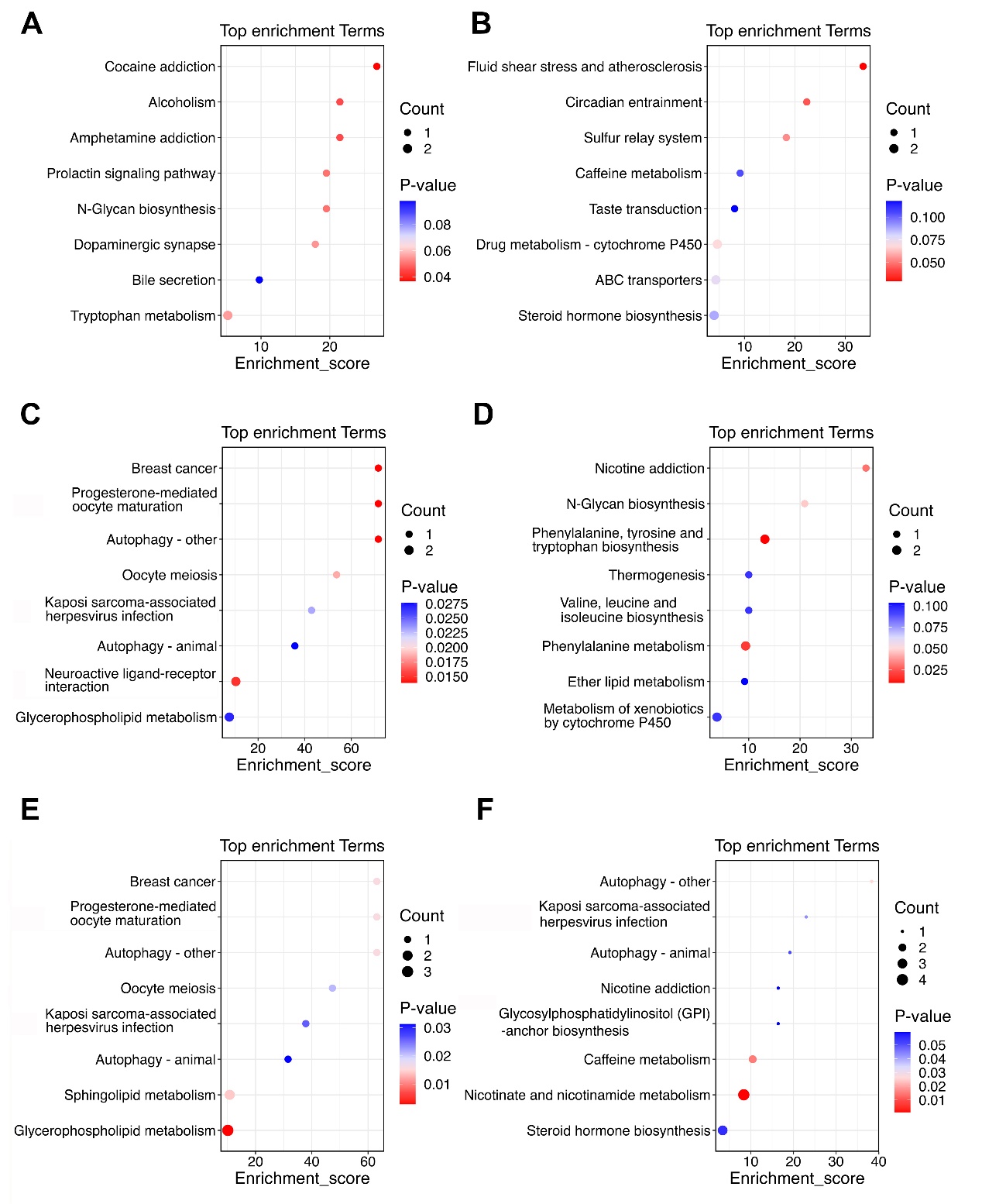


**Supplementary Figure 5. KEGG pathways analysis of different expressed metabolites.** (A) KEGG pathways enriched in upregulated metabolites between OVX and Sham group. (B) KEGG pathways enriched in downregulated metabolites between OVX and Sham group. (C) KEGG pathways enriched in upregulated metabolites between AS_OVX and OVX group. (D) KEGG pathways enriched in downregulated metabolites between AS_OVX and OVX group. (E) KEGG pathways enriched in upregulated metabolites between AS_OVX and Sham group. (F) KEGG pathways enriched in downregulated metabolites between AS_OVX and Sham group.
